# Genetic background and PfKelch13 affect artemisinin susceptibility of PfCoronin mutants in *Plasmodium falciparum*

**DOI:** 10.1371/journal.pgen.1009266

**Published:** 2020-12-28

**Authors:** Aabha I. Sharma, Sara H. Shin, Selina Bopp, Sarah K. Volkman, Daniel L. Hartl, Dyann F. Wirth

**Affiliations:** 1 Department of Immunology and Infectious Diseases, Harvard T.H. Chan School of Public Health, Boston, United States of America; 2 Infectious Disease and Microbiome Program, Broad Institute, Cambridge, United States of America; 3 College of Natural, Behavioral and Health Sciences, Simmons University, Boston, United States of America; 4 Department of Organismic and Evolutionary Biology, Harvard University, Cambridge, United States of America; Michigan State University, UNITED STATES

## Abstract

Malaria continues to impose a significant health burden in the continent of Africa with 213 million cases in 2018 alone, representing 93% of cases worldwide. Because of high transmission of malaria within the continent, the selection pressures to develop drug resistance in African parasites are distinct compared to the rest of the world. In light of the spread of resistance to artemisinin conferred by the C580Y mutation in the *Pf*Kelch13 propeller domain in Southeast Asia, and its independent emergence in South America, it is important to study genetic determinants of resistance in the African context using African parasites. Through *in vitro* evolution of Senegalese parasites, we had previously generated the artemisinin-resistant parasites Pikine_R and Thiès_R and established *pfcoronin* mutations to be sufficient to confer artemisinin resistance in the standard ring-stage survival assay (RSA). In the current study, we used genetic analysis of revertants to demonstrate *pfcoronin* to be the major driver of elevated RSA in the artemisinin-resistant parasites Pikine_R and Thiès_R evolved *in vitro*. We interrogated the role of a second gene *PF3D7_1433800*, which also had mutations in both the Pikine_R and Thiès_R selected lines, but found no evidence of a contribution to reduced susceptibility in the RSA survival assay. Nevertheless, our genetic analysis demonstrates that parasite genetic background is important in the level of *pfcoronin* mediated RSA survival, and therefore we cannot rule out a role for PF3D7_1433800 in other genetic backgrounds. Finally, we tested the potential synergy between the mutations of *pfcoronin* and *pfkelch13* through the generation of single and double mutants in the Pikine genetic background and found that the contribution of *pfcoronin* to reduced susceptibility is masked by the presence of *pfkelch13*. This phenomenon was also observed in the 3D7 background, suggesting that *pfcoronin* may mediate its effects via the same pathway as *pfkelch13*. Investigating the biology of proteins containing the beta-propeller domain could further elucidate the different pathways that the parasite could use to attain resistance.

## Author summary

Artemisinin-based combination therapies remain a crucial standard treatment in combatting malaria around the world. Given the independent emergence of artemisinin resistance associated with *pfkelch13* mutations in Southeast Asia and South America, it is important to study genetic determinants in the African context. Previously, we had generated artemisinin-resistant parasites, referred to as Pikine_R and Thiès_R, by subjecting clinical isolates of parasites from Senegal to artemisinin drug pressure in the laboratory. We found that mutations in the *pfcoronin* gene are sufficient to result in resistance in a standard laboratory assay. Here we report that *pfcoronin* mutations are also necessary for the level of artemisinin resistance observed in both Pikine_R and Thiès_R. In diverse parasite strains the level of resistance is also sensitive to genetic background. Genetic analysis revealed that the resistance phenotype of *pfkelch13* alone masks any additional contribution from *pfcoronin*. Future studies should focus on investigating *pfcoronin* biology.

## Introduction

There were 228 million cases of malaria and over 400,000 malaria deaths worldwide in 2018 [1]. Although significant reductions in disease burden have been achieved since the adoption of artemisinin combination therapies (ACTs) in the early 2000s, the greatest burden of malaria, primarily caused by *Plasmodium falciparum*, continues to occur on the African continent [1]. However, artemisinin drug resistance has emerged in Southeast Asia [2] that is attributed to mutations in the propeller domain of the Kelch13 protein of *P*. *falciparum* (*Pf*Kelch13). The C580Y mutation in particular, which is approaching fixation in Southeast Asia [3], has recently been reported to have independently emerged in South America [4] and Papua New Guinea [5], threatening the success achieved by ACTs. ACTs largely remain effective in the African continent, despite recent reports of *pfkelch13* mutations in Rwanda [6] and Tanzania [7]. Several reports of resistance occurring independently of *pfkelch13*, suggest more than one genetic pathway for resistance [8–10]. Given that the *Pf*Kelch13 C580Y mutation has now been linked to a slowdown of the endocytic machinery for hemoglobin uptake [11], it has yet to be determined if all the different genetic pathways of resistance converge in reducing the uptake of hemoglobin.

The prevalence of malaria, level of immunity, and complexity of infection are very different in the low-transmission setting of Southeast Asia as compared with the African continent. Since Africa has the highest disease burden of *P*. *falciparum* malaria, the selection pressures on African parasites are markedly different from those in Southeast Asia [12,13]. Mutations in *AP2 mu*, encoding the AP-2 complex subunit mu [14], and *ubp1*, encoding the ubiquitin hydrolase, have been linked to artemisinin resistance only in parasites with African genetic backgrounds [9,15]. This observation implies that mechanistic studies of artemisinin resistance should include parasites from different parts of the African continent.

Our interest is therefore in understanding artemisinin resistance in African parasites. Using *in vitro* evolution of resistance, coupled with next generation whole genome sequencing, we previously identified two genes (*PF3D7_1251200 pfcoronin* and *PF3D7_1433800 conserved plasmodium protein of unknown function* [16]) that had mutations in both the independently selected resistant parasites Pikine_R and Thiès_R [17]. No mutations in *pfkelch13* were detected. Mutations in *pfcoronin* G50E (in Thiès_R) and R100K & E107V (in Pikine_R) were of special interest because of the structural similarity of the WD-40 beta-propeller domain of the actin-bundling protein *Pf*Coronin to the beta-propeller domain of *Pf*Kelch13, the known marker of artemisinin resistance in Southeast Asia [2]. Through CRISPR-Cas9 mediated introduction of *pfcoronin* mutations into their respective parental wildtype genetic backgrounds, we established that the *Pf*Coronin mutations are sufficient to confer reduced artemisinin susceptibility as measured by the gold-standard ring-stage survival assay (RSA) [17].

In the work presented here, we further analyzed the role of *pfcoronin* in conferring reduced artemisinin sensitivity in the *in vitro* evolved parasites Thiès_R and Pikine_R. We also studied the role of genetic background in resistance using the standard laboratory strain 3D7. In addition, we investigated the contribution of mutations in *PF3D7_1433800*, which encodes a conserved *Plasmodium* protein of unknown function, and we examined potential phenotypic synergy between *pfcoronin* and *pfkelch13* mutations.

## Results

### *Pf*Coronin is the major driver of reduced susceptibility to artemisinin in the *in vitro* evolved Senegalese parasites

**Fig 1:**
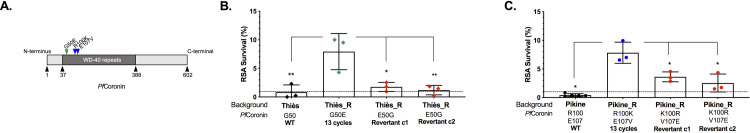
*Pf*Coronin is the major driver of artemisinin resistance in the *in vitro* evolved artemisinin resistant parasites Thiès_R and Pikine_R. **A.** Map of the *Pf*Coronin protein indicating the positions of mutations in the WD-40 beta propeller domain associated with artemisinin resistance (G50E identified in Thiès_R and R100K and E107V identified in Pikine_R). Ring-stage survival assay (RSA) survival percentage for **B.** two clones of the reverted *pfcoronin* G50E mutation in the Thiès_R background (Revertant clone 1 and 2, red), and **C.** two clones of reverted *pfcoronin* R100K and E107V mutations in the Pikine_R background (Revertant clone 1 and 2, red) compared to their respective wildtype (WT) parasites (black). Previously published survivals for Thiès_R (green) and Pikine_R (blue) are included [17]. Each data point represents an independent biological replicate, and the dotted line represents the 1% survival threshold, above which survival is considered resistant. Significance level is indicated by the asterisks, *p<0.05, **p<0.01, determined using an unpaired two-tailed student t-test with Welch’s correction for data in B and Mann-Whitney U test for data in C.

Pikine_R and Thies_R parasites were selected via *in vitro* evolution from parental Senegalese parasites. Each are resistant to artemisinin and carry mutations in the *pfcoronin* gene [17]. Using CRISPR-Cas9 gene editing, we successfully reverted the mutations in *pfcoronin* (Fig 1A) back to the wildtype in both Thiès_R (E50G, S1 Fig) and Pikine_R (K100R & V107E, S2 Fig). When two clones of *pfcoronin* revertants of Thiès_R and the Thiès wildtype (control) were evaluated using RSA and compared to the RSA phenotype of Thiès_R published previously [17] (7.62 ± 2.52%), we found a significant reduction in RSA survival for both clone 1 (1.36 ± 0.61%, *p* = 0.013) and clone 2 (1.19 ± 0.98%, *p* = 0.0098). The Thiès wildtype control had an RSA survival of 0.84 ± 1.22%, *p* = 0.0097 (Fig 1B). Similarly, in the Pikine genetic background, there was a significant reduction in RSA survival for two clones of *pfcoronin* revertants of Pikine_R [clone 1 (3.61 ± 1.33%, *p* = 0.04), clone 2 (2.53 ± 1.66%, *p* = 0.020)]. The Pikine wildtype control had an RSA survival of 0.43 ± 0.27% (*p* = 0.019) compared to the RSA phenotype of Pikine_R published previously [17] (7.83 ± 1.70%) (Fig 1C). All RSA survival values, their corresponding standard deviations, and their sequences confirmed post-RSA, are summarized in S1 and S2 Tables. All the revertant parasites had similar dose-response curves to derivatives of artemisinin as well as control compounds in a 72-hour drug assay (S3 Fig and S3 Table). These results confirmed that the *Pf*Coronin mutations R100K & E107V and G50E are the major drivers of reduced artemisinin sensitivity in our *in vitro* evolved parasites Thiès_R and Pikine_R.

### Parasite background affects RSA survival of *pfcoronin* mutants

**Fig 2:**
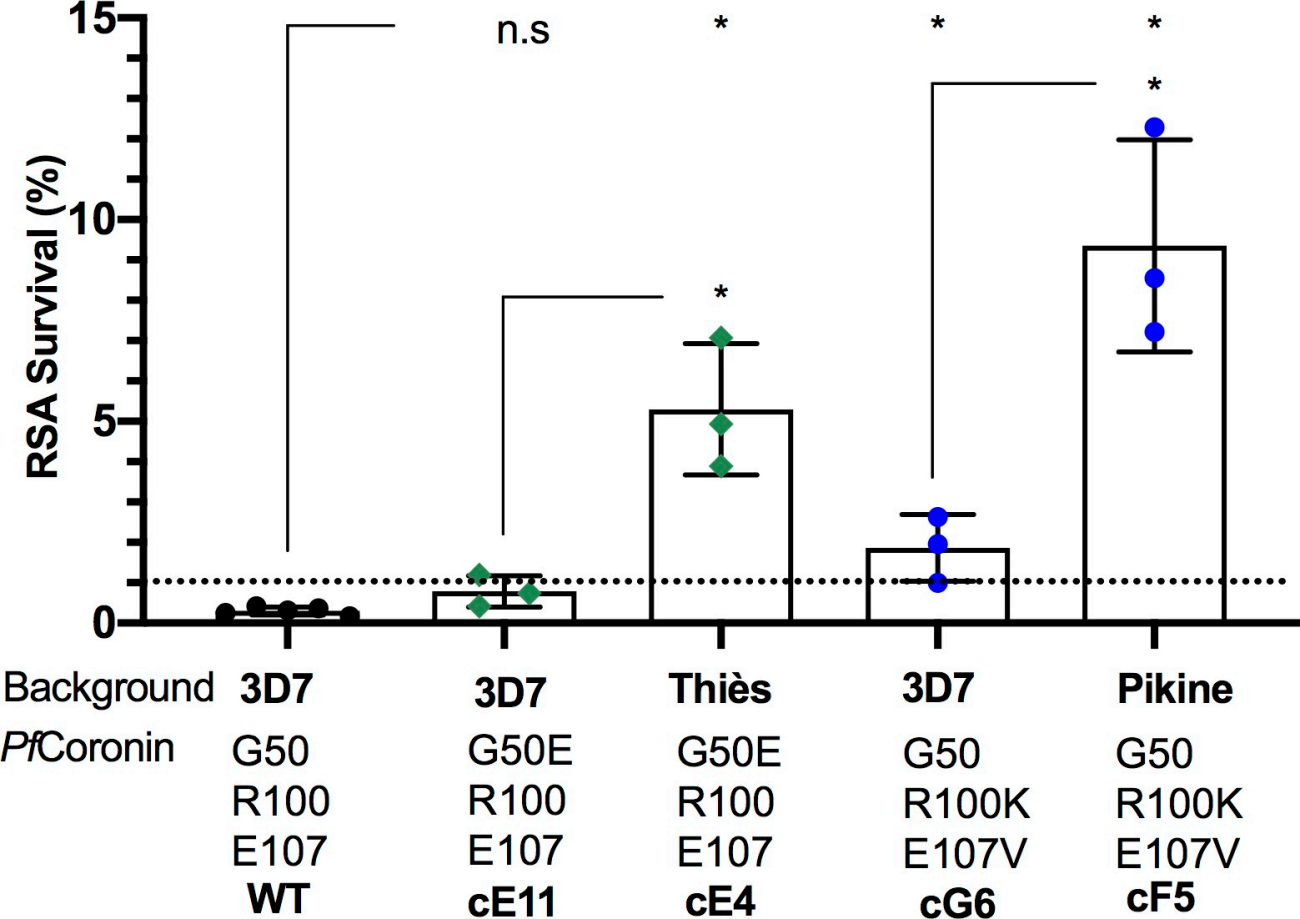
The level of resistance conferred by mutant *pfcoronin* is background dependent. Ring-stage survival assay (RSA) survival percentage of *pfcoronin* mutants in 3D7 background with either G50E (clone E11, green) or R100K and E107V (clone G6, blue) compared to wildtype (WT) 3D7 (black), and previously published [17] survival values for one of the clones of *pfcoronin* CRISPR mutants in the Thiès (clone E4, green) and the Pikine (clone F5, blue) backgrounds. Each data point represents an independent biological replicate and the dotted line represents the 1% survival threshold, above which survival is considered resistant. Significance level is indicated by the asterisks, *p<0.05, determined using an unpaired two-tailed student t-test with Welch’s correction.

To test if RSA survival estimates for *pfcoronin* mutations are affected by the genetic background of the parasite, we generated *pfcoronin* mutants in a 3D7 laboratory parasite and performed RSA (S4 Fig). While the R100K and E107V mutations in *Pf*Coronin were sufficient to pass the 1% RSA survival threshold (1.86 ± 0.86%), which was significantly higher than the 3D7 wildtype (0.30 ± 0.10%, *p* = 0.036), the *Pf*Coronin G50E mutant did not have increased survival (0.79 ± 0.39%, *p* = 0.071) (Fig 2 and S1 and S2 Tables). These RSA survival values are significantly lower than those estimated for *pfcoronin* mutants in Pikine (cF5 survival: 9.35 ± 1.89%, *p* = 0.030) and Thiès (cE4 survival: 5.30 ± 1.25%, *p =* 0.035) genetic backgrounds generated previously using CRISPR/Cas9 gene editing [17] (Fig 2). These findings imply that parasite genetic background is indeed important in the observed level of RSA survival after DHA treatment, even with regard to *pfcoronin* mutations. These results have parallels in the observations for *pfkelch13* when the C580Y mutation was introduced into different parasite backgrounds [18].

### Mutations in *PF3D7_1433800* make a minimal contribution to *in vitro* artemisinin resistance

**Fig 3:**
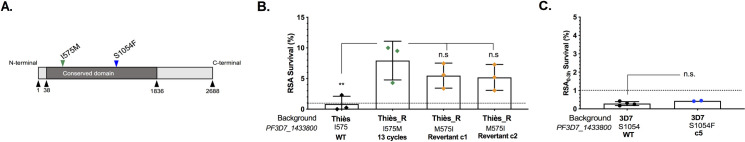
*PF3D7_1433800* makes a minimal contribution to artemisinin resistance. **A.** Map of the conserved *Plasmodium* protein of unknown function (PF3D7_1433800) with the positions for mutations identified in Thiès_R (I575M) and Pikine_R (S1054F) in its conserved domain [16]. **B.** Ring-stage survival assay (RSA) survival percentage for two clones of revertants (M575I, c1 and c2, orange) of *PF3D7_1433800* mutation I575M in Thiès_R compared to our previously published [17] survival for Thiès_R (green) and wildtype (WT) Thiès control (black). **C.** RSA survival percentage is shown for one clone of *PF3D7_1433800* S1054F mutant in the 3D7 background (c5, blue), compared to the WT 3D7 control (black). Each data point represents an independent biological replicate and dotted line represents the 1% survival threshold, above which survival is considered resistant. Significance level is indicated by the asterisks, **p<0.01, determined using an unpaired two-tailed student t-test with Welch’s correction.

We also evaluated the effect on resistance of mutations in the conserved *Plasmodium* protein of unknown function encoded in gene *PF3D7_1433800*, which was the only other gene with mutations in both the independently evolved Thies_R and Pikine_R parasites (I575M in Thiès_R and S1054F in Pikine_R, Fig 3A). This gene has a conserved domain [16] (Fig 3A) and several naturally occurring polymorphisms in both the Thiès and Pikine control parasites (S4 Table), were reported in whole genome sequencing data published previously [17]. Polymorphisms in this gene are found in parasites from all over the world as reported in MalariaGEN [19] (S5 Table). Using a similar approach as that used to test the loss of function as described for *pfcoronin*, we successfully reverted the *PF3D7_1433800* mutation in Thiès_R (I575M) back to the wildtype (M575I) (S5 Fig). The RSA survivals of two clones of *PF3D7_1433800* in Thiès_R were not statistically different (Fig 3) compared with the Thiès_R value published previously [17] (clone 1 survival 5.47 ± 2.97%, *p* = 0.23, clone 2 survival 5.17 ± 3.46%, *p* = 0.19). We also performed growth assays on all the revertants compared to Thiès_R (S6 Fig). There was no significant short-term growth difference for any of the revertant parasites compared to their parent Thiès_R hence differences in RSA survival were not obscured by a difference in growth rate (S6 Fig).

Several attempts to revert the *PF3D7_1433800* mutation S1054F in Pikine_R and introduce S1054F in the Pikine wildtype failed, which made it difficult to evaluate the effect of this gene in the Pikine background. However, we were successful in introducing this mutation in the 3D7 laboratory strain (S7 Fig) to test the gain of function. The *PF3D7_1433800* S1054F mutation in the 3D7 background had survival similar to 3D7 wildtype (Fig 3C), suggesting no evidence of a contribution to reduced sensitivity. Overall, the results from both Thiès_R revertants and 3D7 parasites suggest at most a limited contribution of *PF3D7_1433800* mutations to artemisinin resistance, although we cannot rule out an effect in a different genetic background.

### The phenotypic contributions of mutations in *pfcoronin* and *pfkelch13* are non-additive

**Fig 4:**
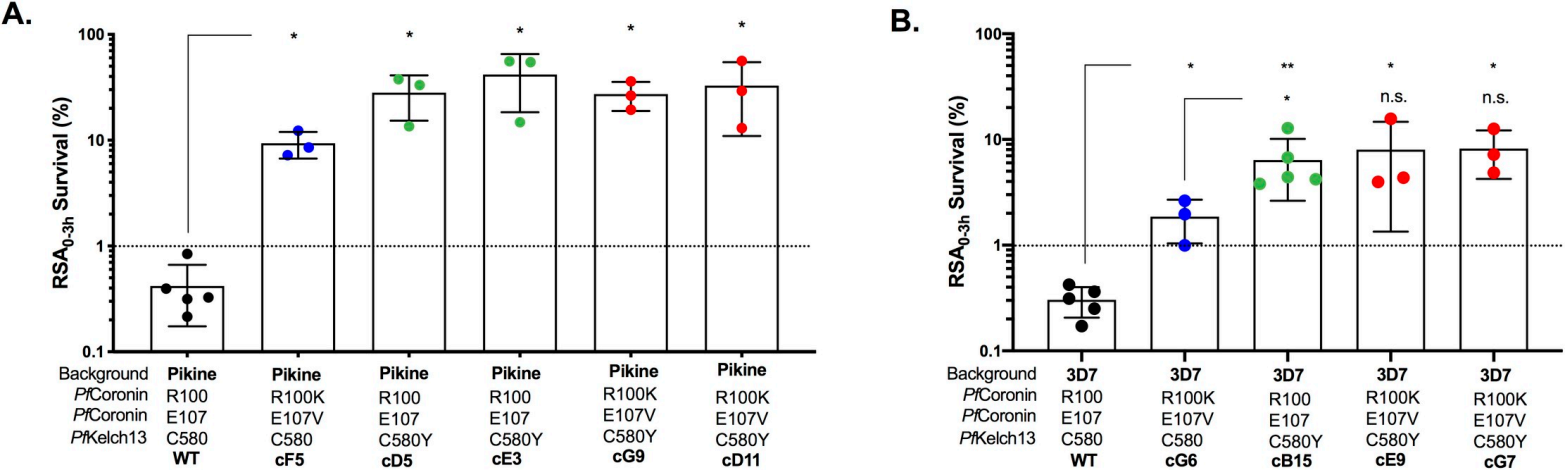
The contributions of *pfcoronin* and *pfkelch13* to resistance are non-additive. **A.** Ring-stage survival assay (RSA) survival percentage for one clone of *pfcoronin* single mutant (cF5 published previously [17], blue), two clones of *pfkelch13* single mutants (cD5 and cE3, green) and two clones of *pfcoronin* and *pfkelch13* double mutants (cG9 and cD11, red) in the Pikine background compared to the wildtype (WT, black). **B.** Ring-stage survival assay (RSA) survival percentage for one clone of *pfcoronin* single mutant (cG6, blue), one clone of *pfkelch13* single mutant (cB15, green) and two clones of *pfcoronin* and *pfkelch13* double mutants (cE9 and cG7, red) in the 3D7 genetic background compared to the wildtype (WT, black). Each point represents an independent biological replicate and dotted line represents the 1% survival threshold, above which survival is considered resistant. Significance level is indicated by the asterisks, *p<005, **p<0.01, determined using two-tailed Mann-Whitney U test.

To test the potential synergy between mutations of *pfcoronin* and *pfkelch13*, we introduced both of these mutations into parasites of either the Pikine or 3D7 genetic background. As a control, we generated *Pf*Kelch13 C580Y parasites (S8B Fig) in the 3D7 background using constructs published previously [20]. Although the C580Y mutation in *Pf*Kelch13 has been investigated in isogenic lines of several different genetic backgrounds [18], it has yet to be studied in West African parasites. We obtained two clones of single mutants of *pfkelch13* and double mutants of *pfkelch13* and *pfcoronin* in the Pikine background parasites (S8 Fig). Two clones of *Pf*Kelch13 C580Y single mutants in Pikine had significantly higher RSA survival values (Fig 4A) compared to the wildtype (wildtype survival: 0.43 ± 0.27%; clone D5 survival: 28.15 ± 12.85%, *p* = 0.036; and clone E3 survival: 41.75 ± 23.39%, *p* = 0.036). The RSA survival for C580Y mutants in the Pikine background was higher than what was reported previously for the single mutants of *pfcoronin* (cF5 survival: 9.35 ± 1.89%%) [17]. The level of RSA survival observed in the double mutants was similar to that of the *pfkelch13* single mutants (Fig 4A) (clone G9 survival: 27.27 ± 8.36%, *p* = 0.036; and clone D11 survival: 32.81 ± 21.83%, *p* = 0.036), which indicates that the phenotypic contribution of *pfcoronin* mutation is masked by the presence of the *pfkelch13* mutation (Fig 4A).

We observed similar results in 3D7 when comparing the RSA phenotype of single and double mutants of *pfkelch13* and *pfcoronin* (Fig 4B). Consistent with previously published work [11,20,21], the C580Y mutant in 3D7 had significantly higher RSA survival (6.38 ± 3.74%) compared with the 3D7 wildtype (0.30 ± 0.10%, *p* = 0.0079) or the 3D7 *Pf*Coronin R100K & E107V single mutant (1.86 ± 0.86%, *p* = 0.036). Double mutants of *pfcoronin* and *pfkelch13* in 3D7 did not show an increased RSA survival when compared to the single C580Y mutation. The RSA survival of the double mutant was significantly higher than the wildtype (clone E9 survival: 8.01 ± 6.67%, *p* = 0.036 and clone G7 survival: 8.20 ± 3.97%, *p* = 0.036). These results are consistent with a non-additive interaction of the two mutations. There were no notable changes in dose-response curves of mutant parasites in the either Pikine or 3D7 genetic backgrounds in a standard 72-hour assay (S3 and S10 Figs and S3 Table).

We tested if RSA phenotype of the single and double mutants in the Pikine genetic background could be explained by a difference in short-term parasite growth of field isolates that grow slower than laboratory strains. We followed the transgenic *pfkelch13* and *pfcoronin* single and double mutants for two reinvasion cycles and compared them to the Pikine wildtype and the *Pf*Coronin R100K & E107V cF5 mutant [17] (S9 Fig). The C580Y single mutants in the Pikine genetic background showed no obvious growth defect. Although the *pfcoronin* single mutant and the double mutants with *pfkelch* and *pfcoronin* had slower apparent growth compared with the wildtype and the *pfkelch13* single mutant, none of the parasite short-term growth curves were significantly different (day 4 parasitemia: 1.40 ± 0.30% for Pikine wildtype, 1.89 ± 0.52% for Pikine C580Y cD5, 2.20 ± 0.55% for Pikine C580Y cE3, 1.76 ± 0.27% for Pikine Coronin R100K and E107V cF5, 1.26 ± 0.17% for double mutant cG9 and 1.56 ± 0.48% for double mutant cD11).

### Naturally occurring polymorphisms of *pfcoronin* and *PF3D7_1433800* occur throughout African countries and Southeast Asia but are distinct from the mutations discovered in Pikine_R or Thiès_R

Using the Pf3K MalariaGEN database of naturally occurring polymorphisms across geographic sites from historic monogenomic samples [19], we examined polymorphisms in *pfcoronin*, *PF3D7_1433800*, and *pfkelch13*. In *pfcoronin*, 37 naturally occurring SNPs were identified, with most of these occurring at a low allele frequency (< 0.1) and in limited geographic locations. Only two polymorphisms, S183G and V424I, had frequencies > 0.1. In contrast to *pfcoronin*, *PF3D7_1433800* is a highly polymorphic gene with 361 SNPs identified across geographic sites (S5 Table), but again most have allele frequencies < 0.1. A subset of sites, particularly in Southeast Asia, have higher allelic frequencies. Analysis of the *pfkelch13* gene identified few polymorphic sites with the exception of the C580Y polymorphism with an allele frequency of 0.7 in Cambodian parasites.

When we compared the naturally occurring polymorphisms to those identified in our *in vitro* selection experiments, none of the nucleotide substitutions or amino acid changes identified in Pikine_R or Thiès_R were present among the naturally occurring SNPs in *pfcoronin* or *PF3D7_1433800*. This observation implies that the mutations occurred *de novo* in the course of selection.

### *pfcoronin* resistance mutations are on the opposite side of actin binding sites in Coronin

**Fig 5:**
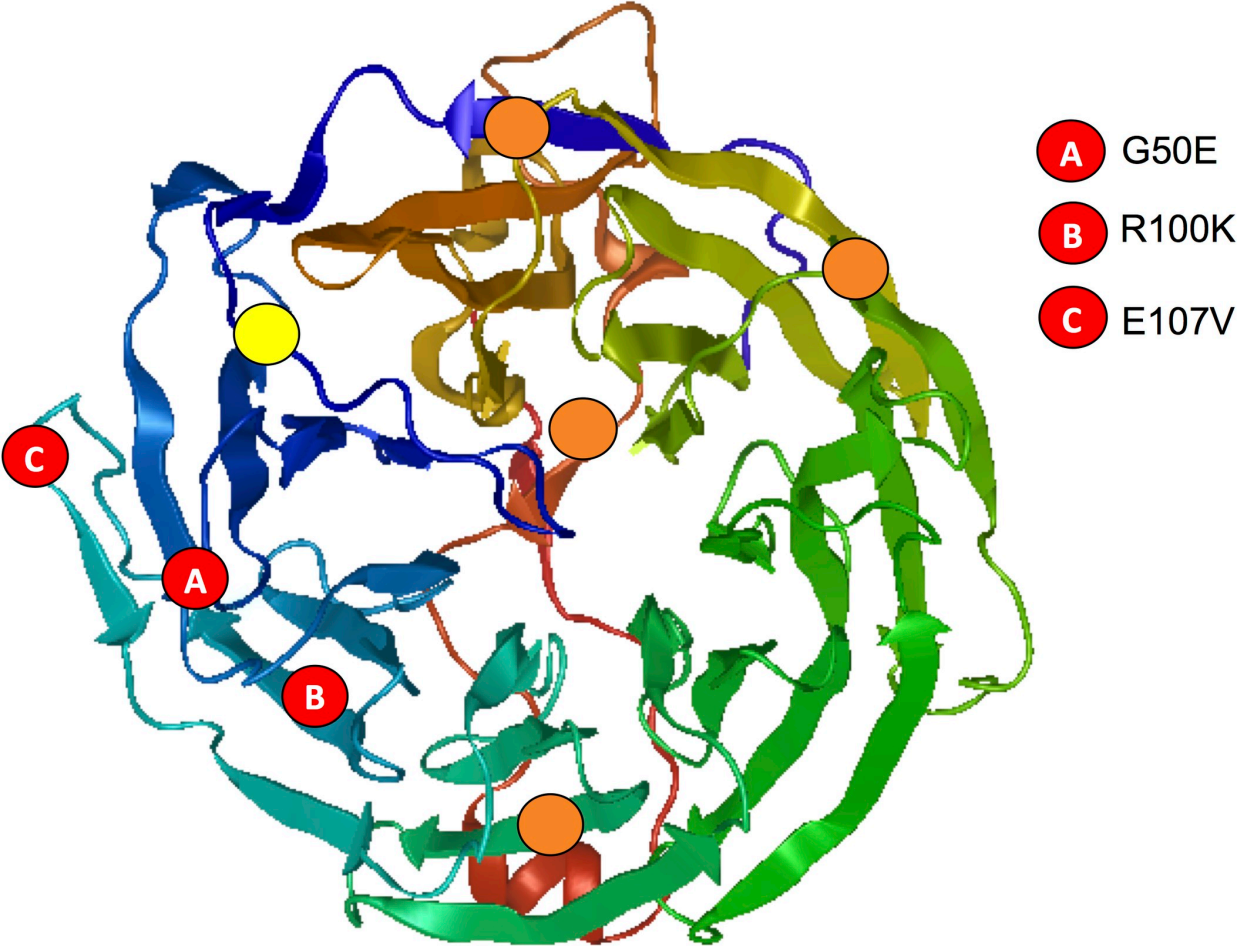
Mutations in *Pf*Coronin conferring resistance to artemisinin are at opposite ends of the putative actin binding sites. Structure of WD-40 domain of *Plasmodium falciparum* generated through SWISS-MODEL homology modeling [22] using the crystal structure of the WD-40 domain of *Toxoplasma gondii* Coronin **(**4OZU.pdb) [24], which is 43% identical with 99% coverage to the the WD-40 domain of *P*. *falciparum*. The predicted actin binding sites are indicated in orange and conserved actin binding site from mouse coronin 1A (R23) [24] indicated in yellow. The mutations that confer artemisinin resistance [17] are indicated in red.

We generated the structure of *Pf*Coronin WD-40 domain through homology modeling [22] using the structurally similar [23] crystal structure of *Toxoplasma gondii* Coronin **(**4OZU.pdb) [24]. When we mapped the mutations in *Pf*Coronin associated with reduced artemisinin susceptibility and the putative actin-binding sites on the structure of the *Pf*Coronin WD-40 domain, the resistance mutations (G50E, R100K and E107V) aligned on the opposite side of most of the putative actin-binding sites (6L-8K, R23, K126-K127, R197 –E201, K282 –D284, L307 –R310, S352 –I353) [24] (Fig 5). The only conserved actin binding site between mouse coronin 1A, *T*. *gondii* Coronin, and *Pf*Coronin is arginine at position 23 (indicated in yellow, Fig 5).

## Discussion

In this study, we established that mutations in *pfcoronin* are the major drivers of reduced artemisinin sensitivity in our *in vitro* evolved Senegalese parasite lines Thiès_R and Pikine_R. When mutations in *pfcoronin* were reverted back to the wildtype in both Thiès_R and Pikine_R, we observed significantly reduced parasite survival in the ring-stage survival assay (RSA) in both genetic backgrounds (Fig 1). This supports our previously published observation of a gain of function associated with the introduction of *pfcoronin* mutations into wildtype parasites [17]. The resistance contribution of *pfcoronin* mutations was dependent on parasite genetic background, consistent with observations made with *pfkelch13* [18]. Although mutations R100K and E107V were sufficient to surpass the 1% threshold of resistance in RSA (Fig 2), the level of resistance for *pfcoronin* mutants was much higher in Senegalese parasites [17] than in the 3D7 laboratory strain. We found no evidence of resistance contribution for mutations in the only other gene, *PF3D7_1433800*, commonly found in the *in vitro* evolved artemisinin resistant parasites (Fig 3). Upon comparison of naturally occurring polymorphisms of this gene and *pfcoronin*, *PF3D7_1433800* was found to be highly polymorphic with 361 non-synonymous mutations found throughout the African region and Southeast Asia (S5 Table). Further work in other African genetic backgrounds will be needed to confirm the lack of a contribution of this gene by itself or in combination with *pfcoronin* mutations. Taken together, our work suggests that mutations in *pfcoronin* are both necessary and sufficient for *in vitro* artemisinin resistance in Pikine_R and Thiès_R, making them the major driver of artemisinin resistance *in vitro*.

We investigated the potential interaction between the mutations in *Pf*Coronin (R100K and E107V) and *Pf*Kelch13 (C580Y) through studies of single and double mutants in both Pikine and 3D7 genetic backgrounds. There was a lack of synergy between the mutations of *Pf*Coronin and *Pf*Kelch13 (Fig 4). In fact, the *pfcoronin* gene was found to be masked by *pfkelch13*. The level of resistance was much higher in the contemporary African parasites compared to laboratory strain 3D7 also of African ancestry, emphasizing the importance of studying genetic factors present using recent clinical isolates. Any potential interaction between *pfcoronin* and *pfkelch13* needs further examination, especially in the African parasites.

The C580Y mutation has recently been linked to reduced expression of *Pf*Kelch13 protein [11,25], resulting in a slowdown of the endocytic machinery for hemoglobin uptake from the host cell without affecting binding interactions of the protein [11]. The artemisinin-resistance phenotype that we observe with *Pf*Coronin could also result from differences in the level of functional protein in the mutants compared to the wildtype. We plan to explore this in the future alongside binding partner interactions of *Pf*Coronin mutants. Considering the role of actin in endocytosis in *Plasmodium* as well as higher eukaryotes [26–28], *Pf*Coronin mediated actin-bundling could be connected to endocytosis mediated artemisinin resistance. Although the resistance mutations are on the opposite side of the putative actin bundling sites (Fig 5), we cannot rule out the potential involvement of the mutations in impairing or aiding actin bundling, which is the major known function of *Pf*Coronin [23,24,29]. It is noteworthy that *T*. *gondii* Coronin has a role in endocytosis and membrane recycling, a process crucial in the endoplasmic reticulum (ER) stress response, independent of its function in actin bundling [24]. This function remains unexplored in *P*. *falciparum* and might suggest an involvement of *Pf*Coronin in the endocytic machinery to attain artemisinin resistance similar to *Pf*Kelch13 without involving actin bundling [11].

WD-40 beta-propeller domains, which are understudied in apicomplexans, are conserved protein structures in eukaryotes that support a wide range of cellular functions [30–33], similar to the beta-propeller domains found in Kelch-like proteins [34]. We still do not know the exact function of *Pf*Kelch13 despite its implication in the ring-stage endocytosis machinery [11], nor do we understand the biological mechanism of artemisinin resistance mediated by *Pf*Coronin. Although widespread resistance-conferring mutations in *pfkelch13* are yet to be reported on the African continent, our *in vitro* work shows that C580Y parasites in the Pikine genetic background exhibit substantial survival in the RSA similar to the Cambodian isolates [2,18,35]. This is unlike Tanzanian F32 parasites which had much lower RSA survival with M476I *pfkelch13* and FCB with C580Y *pfkelch13* [18], raising additional concerns that West African expressing variants of *pfcoronin* and/or *pfkelch13* could be competitive in the field. This emphasizes the need to gather RSA survival estimates for contemporary African parasites. Investigating the biology of beta propeller domain-containing proteins and the possible genetic interactions between different players in artemisinin resistance could further elucidate mechanisms of resistance.

## Methods

### *In vitro* parasite culture

All laboratory strains [3D7 (MR4)], culture-adapted field isolates from Thiès (SenTh032.09) and Pikine (SenP019.04), previously published DHA selected lines Thiès_R and Pikine_R [17], and the subsequently generated CRISPR parasite lines were cultured in O+ red blood cells (RBC) (Interstate Blood Bank (IBB), Memphis, TN) with complete RPMI1640 media (Gibco, Waltham, MA) supplemented with 10% O+ serum (IBB). Parasites were placed in a modular incubator and gassed with 1% O_2_/5% CO_2_/94% N_2_ mixture before incubating at 37°C.

### RSA (Ring-stage Survival Assay)

RSA was performed as described previously [2,35] using 0–3 hour post reinvasion rings that were highly synchronized and exposed to 700 nM DHA or DMSO for 6 hours. Rings were synchronized with 5% D-sorbitol treatment. At 66 hours after the drug washout, parasitemia was assessed by Giemsa-stained smear microscopy of thin smears. The RSA survival percentage was calculated by dividing the parasitemia in DHA-treated parasites over the DMSO-treated control parasitemia. A minimum of two to three biological replicates of RSA were conducted for each parasite line with two technical replicates per biological replicate. Smears were blinded and at least 10,000 RBC counted per replicate.

### Drug sensitivity assays with SYBR Green I

Drug sensitivity assays were performed as previously described [36]. Briefly, ring-stage parasites were grown to 0.8–1% parasitemia in 2% hematocrit in 40μL total volume in 384-well plates. Parasite growth was determined by SYBR Green I staining (Lonza, Visp, Switzerland) of parasite DNA in the trophozoite stage, usually 72 hours after plating. For parasites that grew slowly (double mutants of *pfcoronin* and *pfkelch13* in Pikine background), parasites were stained after 96 hours in culture. The dose-response curves for standard anti-malarial drugs (DHA, ART, MQ; Sigma-Aldrich, St. Louis, MO) were generated from a 12-point dilution series of drugs, carried out in triplicate, centered on expected EC_50_ reported in the literature; with three biological replicates performed for each drug. A SpectraMax M5 (Molecular Devices, Sunnyvale, CA) plate reader was used to measure fluorescence, and data were analyzed with GraphPad Prism version 6 (GraphPad Software, La Jolla, CA). EC_50_ was calculated using nonlinear regression with the log(inhibitor) vs. response with a four-parameter variable slope curve-fitting equation.

### Gene editing using CRISPR-Cas9

Guide sequences targeting the loci of interest were designed using the online tool available via benchling.com. For introducing *pfcoronin* mutations in 3D7 background, we used the strategy successfully implemented previously in Pikine and Thiès parasite backgrounds [17]. For reverting *pfcoronin* G50E, gRNA containing the mutation was used (S1 Fig). We used previously published strategies for reverting *pfcoronin* R100K and E107V, including the gRNA 1 sequence (S2 Fig). All gRNAs were individually annealed and ligated into the BbsI digested pDC2-Cas9-U6-hDHFR plasmid, generously provided by Marcus Lee (Wellcome Sanger Institute, Hinxton, UK), or the pUF1Cas9-U6-DHODH generated using the Cas9 plasmid generated previously [20], both of which contain the U6 snRNA polymerase III promoter and regions for the expression of Cas9 enzyme and human DHFR or yeast DHODH drug selection cassette as described previously [37,38]. For generating *pfkelch13* C580Y mutants, previous constructs [20] were used for the 3D7 background. After several failed transfection attempts with these constructs in the Pikine background, the same gRNA was annealed and ligated into pUF1Cas9-U6-DHODH. Homology regions of about 500bp containing either the SNP of interest or wildtype sequence (Primers used in S6 Table) were cloned into Zeroblunt TOPO vector (Thermo Scientific, Waltham, MA). Site-directed mutagenesis was performed using Quickchange II mutagenesis kit (Agilent, Santa Clara, CA) to scramble guide targeting sequence with shield mutations in the homology region. For C580Y homology region for Pikine background, 500bp homology with SNP and shield mutations was amplified from the construct described previously [20].

Transfection was performed on 5–8% sorbitol synchronized rings from 3D7, SenTh032.09.13.1 (Thiès_R), Sen P019.04 (Pikine wildtype) or SenP019.04.13.1 (Pikine_R) parasites with 50μg of Cas9 plasmid and 50μg of plasmid containing the homology region using the Bio-Rad Gene Pulser at 0.31 kV and 960 μF as described previously [39]. After the transfection, RBCs were plated at 5% hematocrit in complete media. Transfected parasites were allowed to recover for either 8 hours (for 3D7) or overnight (for Senegalese parasite backgrounds) before the addition of 5 nM WR99210 (for pDC2-Cas9-U6-hDHFR plasmid) (Jacobus Pharmaceutical, NJ) or 500nM DSM1 (for pUF1-Cas9-U6-yDHODH plasmid) (Millipore Sigma, Germany). Drug selection was continued for 96 hours for all but the *pfkelch13* mutant generation in Pikine background. For C580Y transfections in Pikine background, drug selection was continued for three weeks. For all transfections, parasite recovery was monitored by microscopy twice a week. Once parasitemia was more than 1%, the loci of interests were genotyped by Sanger sequencing performed by either Psomagen USA (Cambridge, MA) or Genewiz (Cambridge, MA) using the bulk transfectant gDNA. After confirming successful transfection, dilutional cloning was performed in 96-well plate to obtain at least two confirmed CRISPR edited parasite clones.

To confirm the genotype of the edited parasites, parasite DNA was extracted from *in vitro* cultures using QIAamp DNA blood kits (Qiagen, Hilden, Germany), per manufacturer’s instructions. Primers were ordered from Integrated DNA technologies (IDT, Newark, NJ) to amplify relevant candidate gene amplicons of 500–800bp lengths (S6 Table). Primers used for PCR are listed in S6 Table. For the verification of transfectants, primer combinations within and outside of the homology region were used to avoid amplifying the residual plasmid from transfection. PCR amplification was conducted using the Phusion High-Fidelity DNA Polymerase (NEB, Ipswich, MA) following standard procedure. Amplicons were cleaned using ZymoKit DNA purification kit (Irvine, CA) per manufacturer’s instructions and sent for Sanger sequencing to Genewiz.

### Parasite growth assay

Parasite growth assays were conducted over the course of five days. Synchronized rings were plated at 0.5% initial parasitemia and 2% HCT in 6-well plates. Parasitemia was monitored daily using MACSQuant Analyzer Flow Cytometer (Miltenyi Biotec, Germany) through SYBR Green I staining (Lonza, Visp, Switzerland) for 30 minutes at 37°C over the course of five days with two to three biological replicates per parasite line.

### Statistical analysis

All statistical analyses were first performed in groups of two using parametric unpaired two-tailed t-test with Welch’s correction. If this resulted in a significantly different F test to compare variances for the pair, making the results of the Welch’s correction unreliable, a non-parametric Mann-Whitney test was conducted using GraphPad Prism version 6.

### *Pf*Coronin homology modeling

*Pf*Coronin homology modeling was generated using SWISS-MODEL automated server [22] using *Toxoplasma gondii* Coronin crystal structure (4ozu.1) [24] (43.26% identity, 99% coverage, 0.77 GMQE, -2.49 QMEAN) was downloaded and visualized using Protean 3D software Version 12.0 (DNASTAR package, Madison, WI).

## Supporting information

S1 Fig **A.** CRISPR gene editing strategy for generating *pfcoronin* revertants in the SenTh032.09.13.1 (Thiès_R) background. Homology region with primer sequences underlined, *pfcoronin* mutated site indicated in red, shield mutations in green, protospacer adjacent motif (PAM) sequences highlighted in yellow. **B.** Sanger sequencing confirmation of CRISPR edited parasite gDNA highlighting the target SNP in red and shield mutations in green compared to the parent and Thiès_R.
(DOCX)

S2 Fig **A.** CRISPR gene editing strategy for generating *pfcoronin* revertants in the SenP019.04.13.1 (Pikine_R) background. Homology region with primer sequences underlined, *pfcoronin* mutated sites indicated in red, shield mutations in green, protospacer adjacent motif (PAM) sequences highlighted in yellow. **B.** Sanger sequencing confirmation of CRISPR edited parasite gDNA highlighting the target SNPs in red and shield mutations in green compared to the parent and Pikine_R.
(DOCX)

S3 Fig EC_50_ values in response to artemether (AM), dihydroartemisinin (DHA) or artemisinin (ART) and control compound mefloquine (MQ) are unchanged in clones of single (*Pf*Kelch13 C580Y) and double mutants (*Pf*Kelch13 C580Y and *Pf*Coronin R100K & E107V) in Pikine background and revertant clones in Pikine_R and Thiès_R. Parasite drug sensitivity was measured by 72-hour *in vitro* assays with SYBR Green. For parasites that grew slowly (double mutants of *pfcoronin* and *pfkelch13* in the Pikine background), parasites were stained after 96 hours in culture. Representative EC_50_ dose-response curves from three biological replicates are presented.
(TIFF)

S4 Fig Sanger sequencing confirmation of CRISPR edited gDNA highlighting the target SNP in red and shield mutations in green of *Pf*Coronin A. G50E mutation corresponding to clone E11 and **B.** R100K/E107V mutations corresponding to clone G6 compared to 3D7 wildtype.
(DOCX)

S5 Fig **A.** CRISPR gene editing strategy for generating PF*3D7_1433800* revertants in the SenTh032.09.13.1 (Thiès_R) background. Homology region with primer sequences underlined, *PF3D7_1433800* mutated site indicated in red, shield mutations in green, protospacer adjacent motif (PAM) sequences highlighted in yellow. **B.** Sanger sequencing confirmation of CRISPR edited parasite gDNA highlighting the target SNP in red and shield mutations in green compared to the parent and Thiès_R.
(DOCX)

S6 Fig Revertants of *pfcoronin* and *PF3D7_1433800* in Thiès background have no short-term growth defect. Normalized growth curve from two biological replicates of Thiès background parasites referred to in main Figs 1B and 3B followed through two reinvasion cycles for growth comparison. Statistical analyses presented no significant difference in growth for any of the parasites.
(TIFF)

S7 Fig **A.** CRISPR gene editing strategy for generating PF*3D7_1433800* S1054F in the 3D7 background after several attempts to knock-in or revert the SNP failed in SenPik19.04 (Pikine background parasite). Homology region with primer sequences underlined, *PF3D7_1433800* mutated site indicated in red, shield mutations in green, protospacer adjacent motif (PAM) sequences highlighted in yellow. One of the shield mutations modified the PAM sequence. **B.** Sanger sequencing confirmation of CRISPR edited clonal parasite gDNA highlighting the target SNP in red and shield mutations in green compared to the parent. The region is highly AT rich resulting in high Sanger sequencing background.
(DOCX)

S8 Fig Sanger sequencing confirmation of CRISPR mutants generated using previously published constructs. **A.** Confirmation of *pfcoronin* editing to generate R100K & E107V mutants in 3D7 background using constructs described previously for the Pikine background^1^. **B.** Confirmation of *pfkelch13* editing to generate C580Y mutants in both 3D7 and Pikine backgrounds. Target SNP(s) are highlighted in red and shield mutations in green and are compared to the wildtype (WT) sequence.
(DOCX)

S9 Fig Single and double mutants of *pfcoronin* and *pfkelch13* in the Pikine background have no short-term growth delay. Normalized growth curve from three biological replicates of Pikine background parasites from Fig 4A followed through two reinvasion cycles for growth comparison. Statistical analyses presented no significant difference in growth for any of the parasites.
(TIFF)

S10 Fig EC_50_ in response to artemisinin derivatives artemether (AM), dihydroartemisin (DHA) and control compound mefloquine (MQ) are unchanged for various mutants in 3D7 background. Parasite drug sensitivity was measured by 72-hour *in vitro* assays with SYBR Green (See methods for details). Representative EC_50_ dose-response curves from three biological replicates are presented.
(TIFF)

S1 Table Summary of RSA values for all parasite lines. (DOCX)

S2 Table Sequencing confirmation of parasite lines post-RSA. Parasites were sequenced from DMSO treated group after RSA was completed. Representative chromatogram from one of the biological replicates is presented. SNP and shield mutation positions are shown in red and green, respectively, for CRISPR edited parasites as well as their respective WT sequences.
(DOCX)

S3 Table Summary of IC50 values for artemether (AM), dihydroartemisinin (DHA) and mefloquine (MQ) for all parasite lines. (DOCX)

S4 Table *PF3D7_1433800* conserved protein of unknown function and its background mutations in Senegalese parasites Pikine and Thiès. (DOCX)

S5 Table **Geographical distribution of naturally occurring non-synonymous polymorphisms of *pfcoronin, PF3D7_1433800* and *pfkelch13* in African countries (green columns) and Southeast Asia (orange columns) based on PF3K database**.
(XLSX)

S6 Table PCR primers to amplify *pfcoronin* (*PF3D7_1251200*), *pfkelch13* (*PF3D7_1343700*) and *PF3D7_1433800* conserved protein of unknown function. For mutagenesis (mut) primers, shield mutations are highlighted in green and targeted mutation(s), if present within the range of the primer, are highlighted in red.
(DOCX)
