# Supplementary material for: Genetic background and PfKelch13 affect artemisinin susceptibility of PfCoronin mutants in Plasmodium falciparum
Source: PLoS Genet. 2020 Dec 28;16(12):e1009266. doi: 10.1371/journal.pgen.1009266 (PMC7793257; doi:10.1371/journal.pgen.1009266)

1. Homology region for *Pf*Coronin E50G revertant replacement: 501bp

GTATAGCTTGTAGTGCTGGATATATTGCTgtaaggaaaaaaaaaaaaaataataataataaaaaattaaaatgtacaaatgatgcagtatatatgtatatgtatttatgtatatatttatgtatatatgtatgtacatatttatgtatgtatatatttggtgcgctttaaagattaattcattattcatatatatcgttttatatgatttgttcatattatagGTACCATGGCAAGTTGAGGGTGGAGGAATGATCGGAGTTATCAGATTAGAAAATCAAGTGAGAAATCCCCCTGTAATAAAATTGAAGAGTCATACATCTCCCATCCTTeGATTTGTCATTTAACCCGTGTTATAGTGAGATATTAGCTTCATGTTCAGAAGATATGTCTATAAGAATATGGGAGATACGTCATGAGGATGAGAATGTGAATGAGGTAAAGGATCCTTTATGTATATTAAATGGTCATAAGAAAAAAGTAAATATATTATCATGGAATCC

Guide RNA for Cas9 plasmid

Guide 15’CCATGGCAAGTTGAAGGGGT

WT AA Sequence:     VPWQVEGGGMIGVIRLEN

Thiès_R AA Sequence:  VPWQVEGEGMIGVIRLEN

Reverted AA Sequence: VPWQVEGGGMIGVIRLEN

**B.**


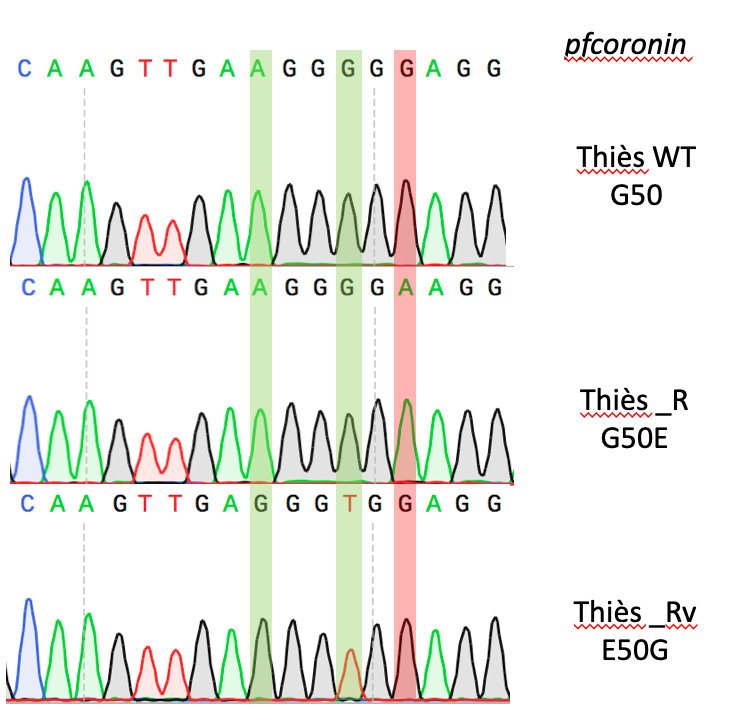

Supplement: S1 Fig — A. CRISPR gene editing strategy for generating pfcoronin revertants in the SenTh032.09.13.1 (Thiès_R) background. Homology region with primer sequences underlined, pfcoronin mutated site indicated in red, shield mutations in green, protospacer adjacent motif (PAM) sequences highlighted in yellow. B. Sanger sequencing confirmation of CRISPR edited parasite gDNA highlighting the target SNP in red and shield mutations in green compared to the parent and Thiès_R. (DOCX) [file pgen.1009266.s001.docx]
