# Supplementary material for: Genetic background and PfKelch13 affect artemisinin susceptibility of PfCoronin mutants in Plasmodium falciparum
Source: PLoS Genet. 2020 Dec 28;16(12):e1009266. doi: 10.1371/journal.pgen.1009266 (PMC7793257; doi:10.1371/journal.pgen.1009266)

1. Homology region for *Pf*Coronin K100R V107E revertant replacement: 500bp

ggtgcgctttaaagattaattcattattcatatatatcgttttatatgatttgttcatattatagGTACCATGGCAAGTTGAAGGGGGAGGAATGATCGGAGTTATCAGATTAGAAAATCAAGTGAGAAATCCCCCTGTAATAAAATTGAAGAGTCATACATCTCCCATCCTTGATTTGTCATTTAACCCGTGTTATAGTGAGATATTAGCTTCATGTTCAGAAGATATGTCTATAAGAATATGGGAGATTCGACATGAGGATGAGAATGTGAATGAGGTAAAGGATCCTTTATGTATATTAAATGGTCATAAGAAAAAAGTAAATATATTATCATGGAATCCTATGAATTATTTTATATTATCATCTACCTCTTTTGATTCTTCTGTTAATATATGGGATATAGAAAATGAGAAGAAAGCCTTTGAAATAAATATGCCAAAGAAATTAAGTTCTTTACAATGGGATATCGGTGGTAATTTATTAAGTGGAACTTGTCAG

Guide RNA for Cas9 plasmid

Guide 15’AAATATGGGAGATACGTCATG

WT AA Sequence:        SEDMSIRIWEIRHEDENVNEV

Pikine_R AA Sequence:  SEDMSIKIWEIRHVDENVNEV

Reverted AA Sequence:SEDMSIRIWEIRHEDENVNEV


 
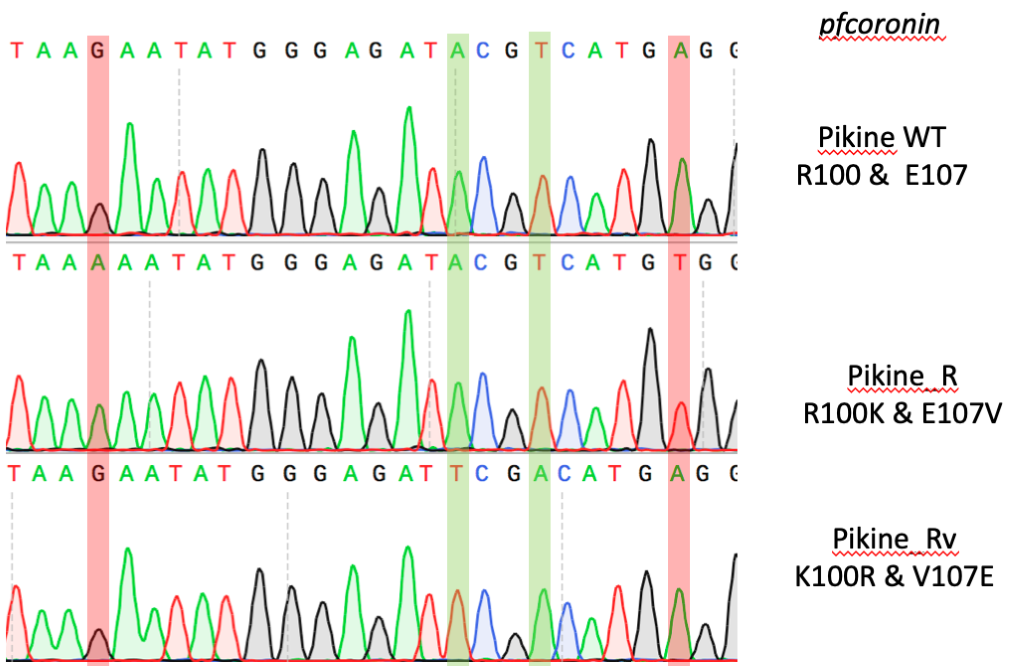

Supplement: S2 Fig — A. CRISPR gene editing strategy for generating pfcoronin revertants in the SenP019.04.13.1 (Pikine_R) background. Homology region with primer sequences underlined, pfcoronin mutated sites indicated in red, shield mutations in green, protospacer adjacent motif (PAM) sequences highlighted in yellow. B. Sanger sequencing confirmation of CRISPR edited parasite gDNA highlighting the target SNPs in red and shield mutations in green compared to the parent and Pikine_R. (DOCX) [file pgen.1009266.s002.docx]
