# Supplementary figures and images for: Genetic background and PfKelch13 affect artemisinin susceptibility of PfCoronin mutants in Plasmodium falciparum
Source: PLoS Genet. 2020 Dec 28;16(12):e1009266. doi: 10.1371/journal.pgen.1009266 (PMC7793257; doi:10.1371/journal.pgen.1009266)

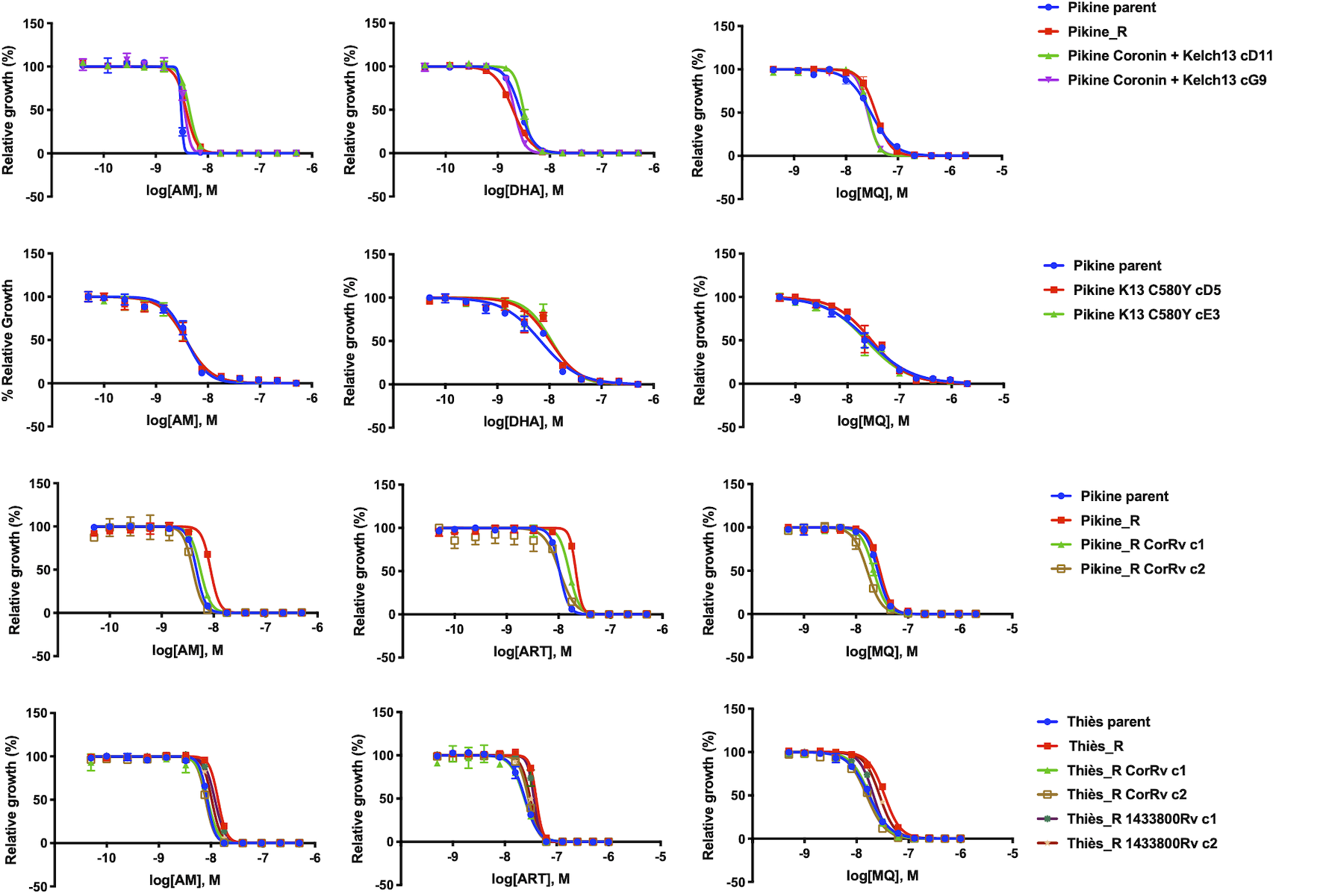

Supplement: S3 Fig — Parasite drug sensitivity was measured by 72-hour in vitro assays with SYBR Green. For parasites that grew slowly (double mutants of pfcoronin and pfkelch13 in the Pikine background), parasites were stained after 96 hours in culture. Representative EC50 dose-response curves from three biological replicates are presented. (TIFF) [file pgen.1009266.s003.tiff]

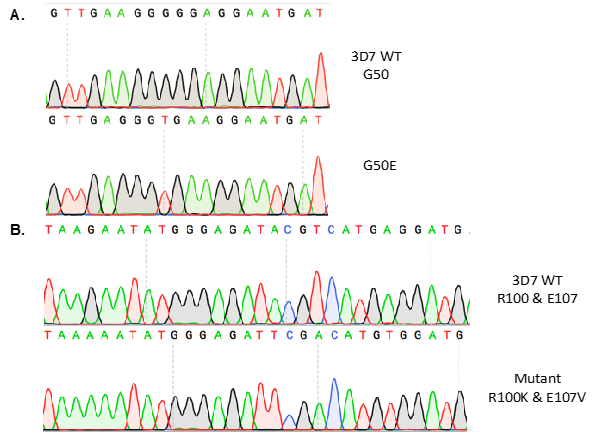

Supplement: S4 Fig — G50E mutation corresponding to clone E11 and B. R100K/E107V mutations corresponding to clone G6 compared to 3D7 wildtype. (DOCX) [file pgen.1009266.s004.docx]

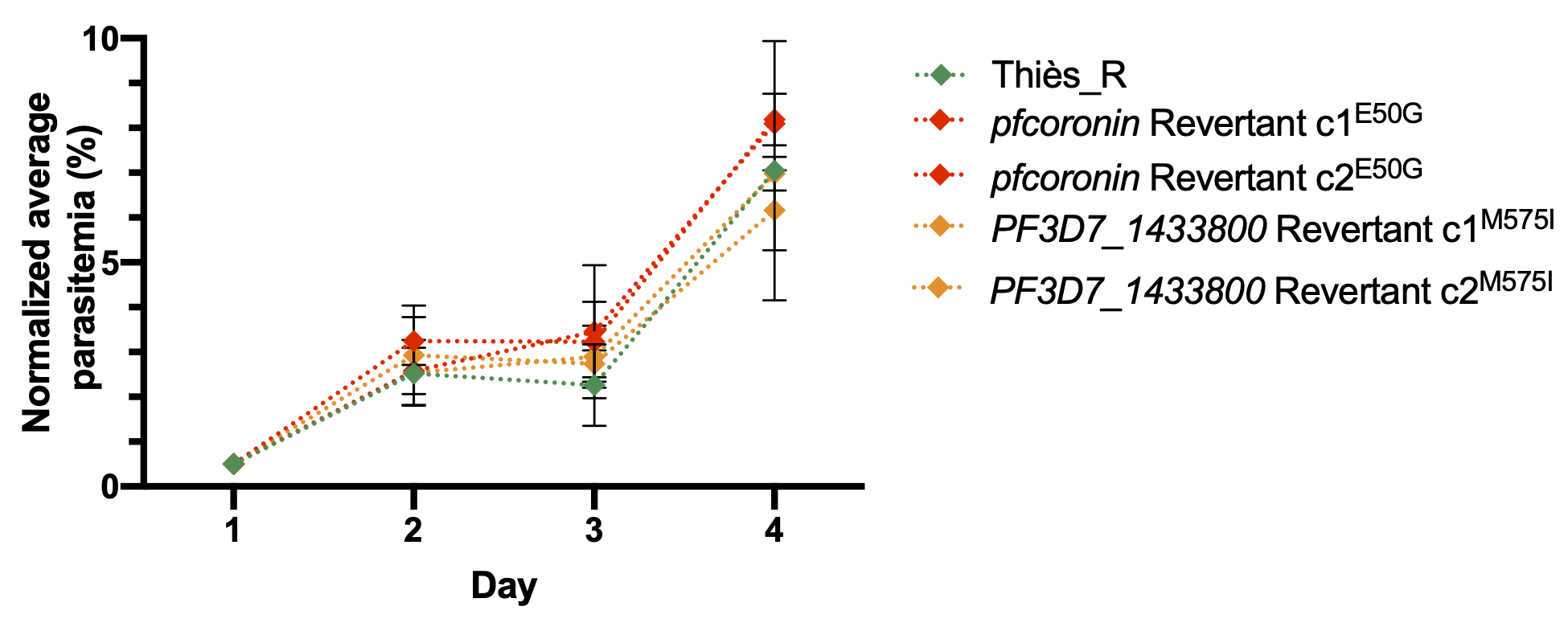

Supplement: S6 Fig — Normalized growth curve from two biological replicates of Thiès background parasites referred to in main Figs 1B and 3B followed through two reinvasion cycles for growth comparison. Statistical analyses presented no significant difference in growth for any of the parasites. (TIFF) [file pgen.1009266.s006.tiff]

1.
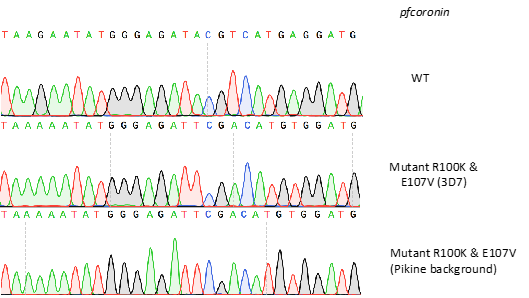


1.
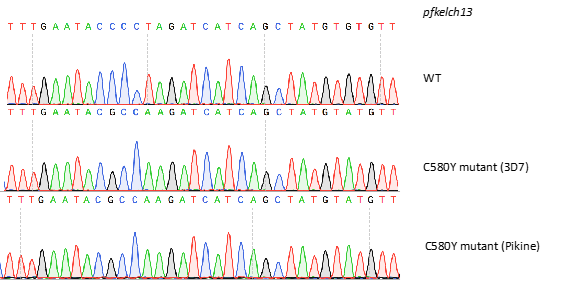

Supplement: S8 Fig — A. Confirmation of pfcoronin editing to generate R100K & E107V mutants in 3D7 background using constructs described previously for the Pikine background1. B. Confirmation of pfkelch13 editing to generate C580Y mutants in both 3D7 and Pikine backgrounds. Target SNP(s) are highlighted in red and shield mutations in green and are compared to the wildtype (WT) sequence. (DOCX) [file pgen.1009266.s008.docx]

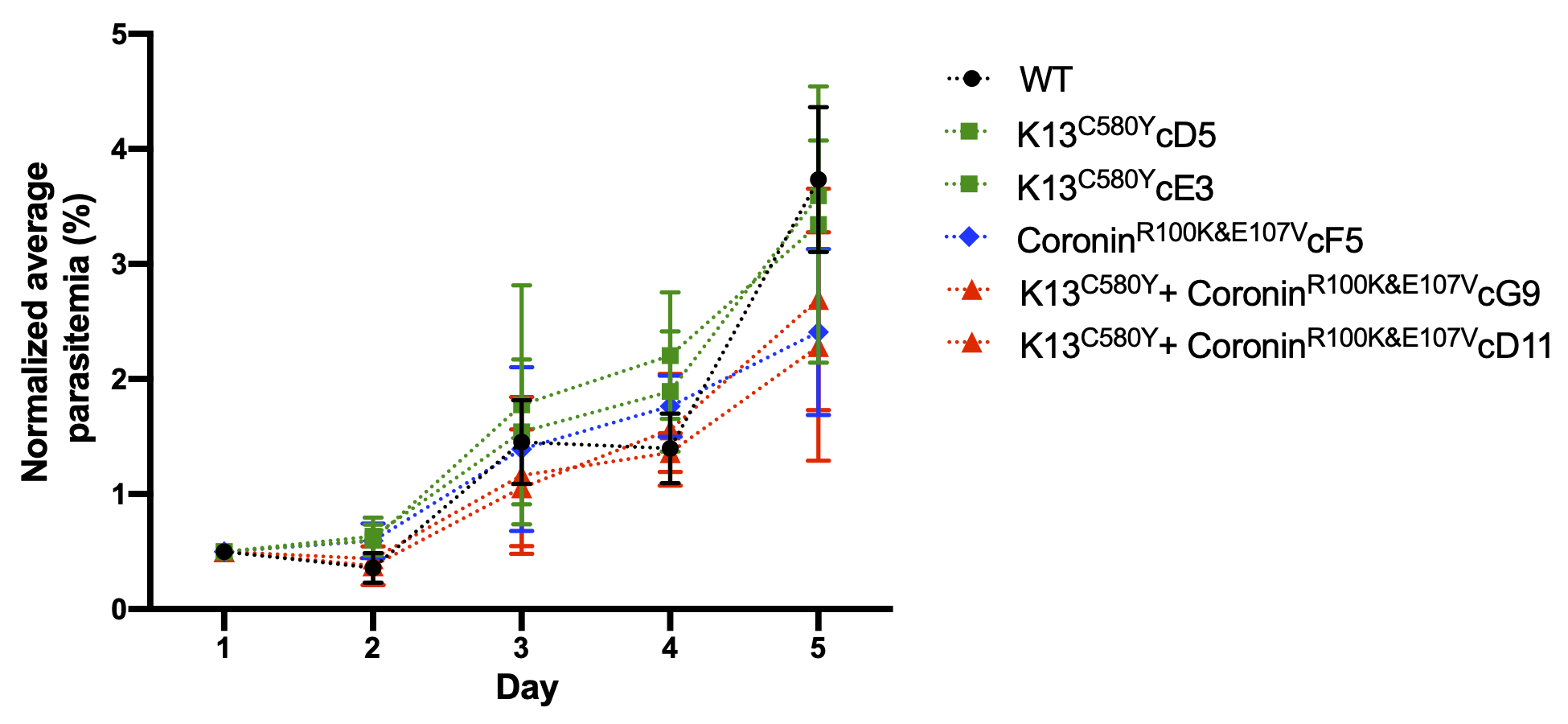

Supplement: S9 Fig — Normalized growth curve from three biological replicates of Pikine background parasites from Fig 4A followed through two reinvasion cycles for growth comparison. Statistical analyses presented no significant difference in growth for any of the parasites. (TIFF) [file pgen.1009266.s009.tiff]

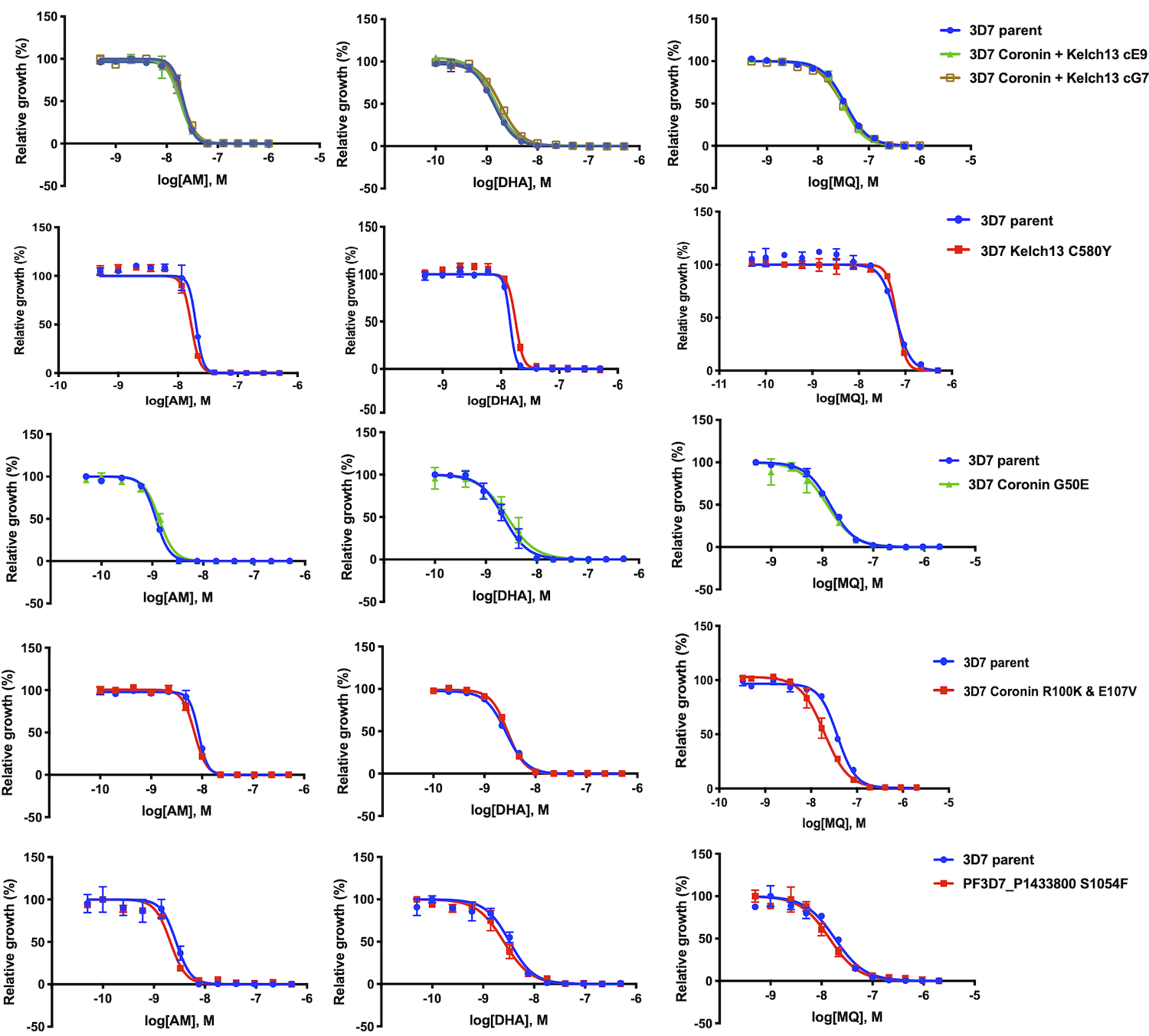

Supplement: S10 Fig — Parasite drug sensitivity was measured by 72-hour in vitro assays with SYBR Green (See methods for details). Representative EC50 dose-response curves from three biological replicates are presented. (TIFF) [file pgen.1009266.s010.tiff]
