# Supplementary material for: Genetic background and PfKelch13 affect artemisinin susceptibility of PfCoronin mutants in Plasmodium falciparum
Source: PLoS Genet. 2020 Dec 28;16(12):e1009266. doi: 10.1371/journal.pgen.1009266 (PMC7793257; doi:10.1371/journal.pgen.1009266)

1. Homology region for S1054F SNP replacement: 451bp

GATGAATAATAATATGTTTTATGATAATGGTTTAAATTATAATATGTTGGACAATAATACATTTAATGGTAATATTTTAAATAATAACCAAATAATAAAATGTAATAAAAGTACGATTCATAAATTAATAAGGAATGAAAAAACGGATATATATAAACATGTGTCTATATACAAATTTTCATCAAACCTTATAAATATACAACATAATTTATTTAATATATATTTGGAATGTAATTTATTAAATAGAGCTTATGAACTTGTCATGTCAGATTTTGATTTTTTTTTTTCATTAACAGAAGAAATAATTTTCTTATATAAAATATATTTAATTGAATTGAAAAAAAAAAACATTTTGCATTCTTTTGAAATATTAAATATAGCTTTTGATAAATGTTATGTTTTGTTAAAGAAATATTTATGTAATCCAACATCATTTAAATTATTATCAG

Guide RNA for Cas9 plasmid

Guide 15’ GTACAACTTAACGTTTTCCA

WT AA Sequence:     AYELVMSDFDFSFSLTEE

Mutated AA Sequence:  AYELVMSDFDFFFSLTEE


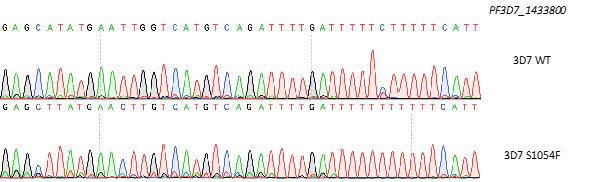

Supplement: S7 Fig — A. CRISPR gene editing strategy for generating PF3D7_1433800 S1054F in the 3D7 background after several attempts to knock-in or revert the SNP failed in SenPik19.04 (Pikine background parasite). Homology region with primer sequences underlined, PF3D7_1433800 mutated site indicated in red, shield mutations in green, protospacer adjacent motif (PAM) sequences highlighted in yellow. One of the shield mutations modified the PAM sequence. B. Sanger sequencing confirmation of CRISPR edited clonal parasite gDNA highlighting the target SNP in red and shield mutations in green compared to the parent. The region is highly AT rich resulting in high Sanger sequencing background. (DOCX) [file pgen.1009266.s007.docx]
