# Supplementary material for: Genetic background and PfKelch13 affect artemisinin susceptibility of PfCoronin mutants in Plasmodium falciparum
Source: PLoS Genet. 2020 Dec 28;16(12):e1009266. doi: 10.1371/journal.pgen.1009266 (PMC7793257; doi:10.1371/journal.pgen.1009266)
Supplement: S1 Table — (DOCX) [file pgen.1009266.s011.docx]

| **Background** | **Protein** | **Mutation** | **Clone name** | **Biorep 1 RSA_0-3h_ survival (%)** | **Biorep 2* RSA_0-3h_ survival (%)** | **Biorep 3* RSA_0-3h_ survival (%)** | **Mean RSA_0-3h_ survival (%)** | **Standard deviation**  **(SD)** |
| --- | --- | --- | --- | --- | --- | --- | --- | --- |
| Pikine |  | Wildtype |  | 0.21 | 0.84, 0.32 | 0.39, 0.32 | 0.43 | 0.27 |
| Pikine | *Pf*Kelch13 | C580Y | cD5 | 37.71 | 13.54 | 33.21 | 28.15 | 12.85 |
| Pikine | *Pf*Kelch13 | C580Y | cE3 | 55.80 | 14.75 | 54.70 | 41.75 | 23.39 |
| Pikine | *Pf*Coronin & *Pf*Kelch13 | R100K, E107V & C580Y | cG9 | 19.37 | 26.41 | 36.02 | 27.27 | 8.36 |
| Pikine | *Pf*Coronin & *Pf*Kelch13 | R100K, E107V & C580Y | cD11 | 13.00 | 29.20 | 56.22 | 32.81 | 21.83 |
| Pikine_R | *Pf*Coronin | K100R, V107E | Revertant c1 | 3.46 | 2.84 | 4.54 | 3.61 | 1.33 |
| Pikine_R | *Pf*Coronin | K100R, V107E | Revertant c2 | 1.75 | 1.51 | 4.34 | 2.53 | 1.66 |
| Thiès |  | Wildtype |  | 0.00 | 0.24 | 2.28 | 0.84 | 1.22 |
| Thiès_R | *Pf*Coronin | E50G | Revertant c1 | 1.78 | 0.04 | 2.53 | 1.36 | 0.61 |
| Thiès_R | *Pf*Coronin | E50G | Revertant c2 | 0.26 | 1.47 | 1.83 | 1.19 | 0.98 |
| Thiès_R | PF3D7_1433800 | M575I | Revertant c1 | 3.36 | 5.59 | 7.45 | 5.47 | 2.97 |
| Thiès_R | PF3D7_1433800 | M575I | Revertant c2 | 3.06 | 7.28 | 5.20 | 5.17 | 3.46 |
| 3D7 |  | Wildtype |  | 0.17 | 0.31,0.25 | 0.42, 0.36 | 0.30 | 0.10 |
| 3D7 | *Pf*Coronin | R100K, E107V | cG6 | 2.63 | 1.96 | 0.99 | 1.86 | 0.86 |
| 3D7 | *Pf*Coronin | G50E | cE11 | 0.73 | 0.42 | 1.19 | 0.79 | 0.39 |
| 3D7 | *Pf*Kelch13 | C580Y | cB15 | 4.40 | 3.81, 4.20 | 6.73, 12.77 | 6.38 | 3.74 |
| 3D7 | PF3D7_1433800 | S1054F | c5 | 0.42 | 0.45 |  | 0.43 | 0.02 |
| 3D7 | PfCoronin & *Pf*Kelch13 | R100K, E107V & C580Y | cE9 | 4.35, | 3.97 | 15.70 | 8.01 | 6.67 |
| 3D7 | *Pf*Coronin & *Pf*Kelch13 | R100K, E107V & C580Y | cG7 | 7.18 | 4.85 | 12.58 | 8.20 | 3.97 |

* Extra bioreps accommodated in Biorep 2 and 3 columns if available
