# Supplementary material for: Genetic background and PfKelch13 affect artemisinin susceptibility of PfCoronin mutants in Plasmodium falciparum
Source: PLoS Genet. 2020 Dec 28;16(12):e1009266. doi: 10.1371/journal.pgen.1009266 (PMC7793257; doi:10.1371/journal.pgen.1009266)
Supplement: S2 Table — Parasites were sequenced from DMSO treated group after RSA was completed. Representative chromatogram from one of the biological replicates is presented. SNP and shield mutation positions are shown in red and green, respectively, for CRISPR edited parasites as well as their respective WT sequences. (DOCX) [file pgen.1009266.s012.docx]

| **Background** | **Protein** | **Mutation** | **Clone name** | **Chromatogram** |
| --- | --- | --- | --- | --- |
| Pikine |  | Wildtype |  | C580  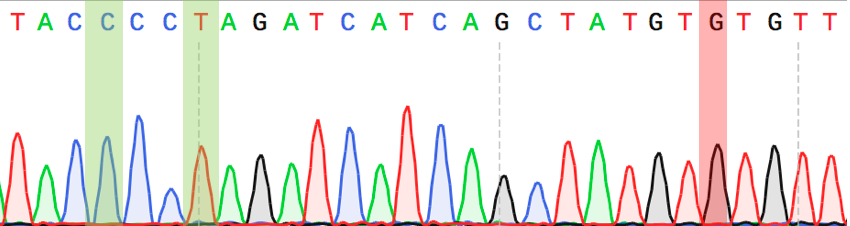      R100, E107  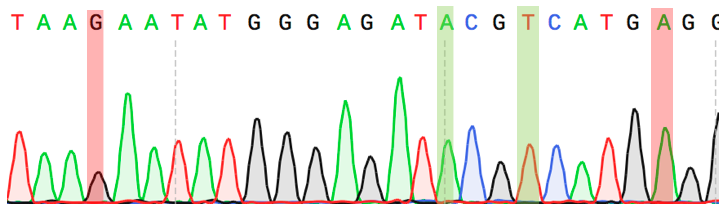 |
| Pikine | *Pf*Kelch13 | C580Y | cD5 | 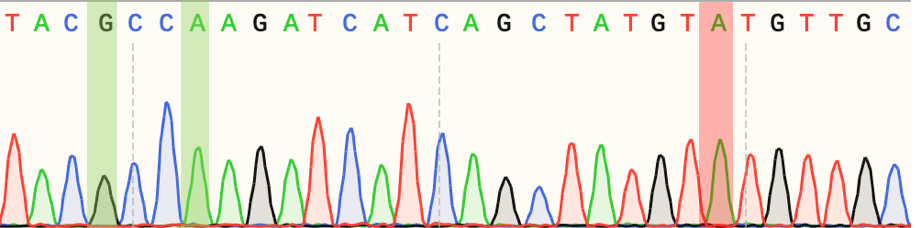 |
| Pikine | *Pf*Kelch13 | C580Y | cE3 | 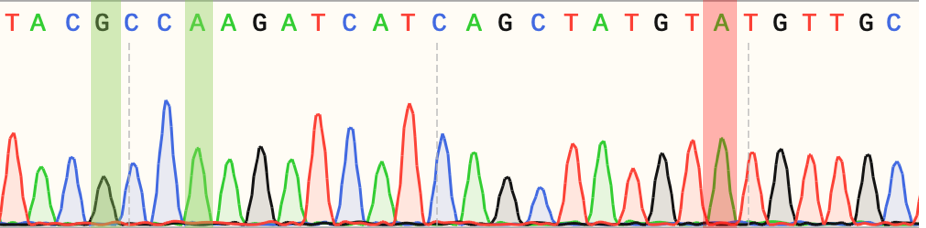 |
| Pikine | *Pf*Coronin & *Pf*Kelch13 | R100K, E107V & C580Y | cG9 | C580Y  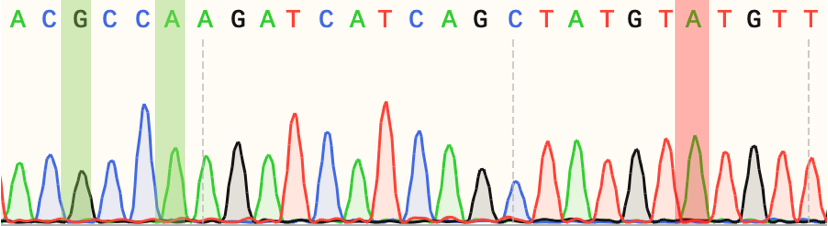    R100K, E107V   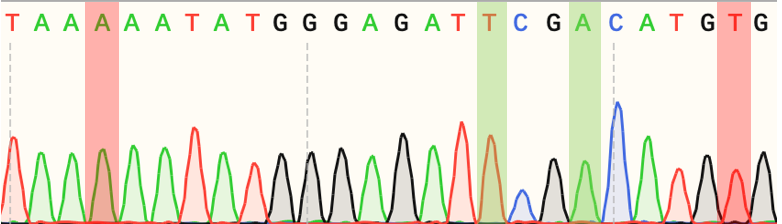 |
| Pikine | *Pf*Coronin & *Pf*Kelch13 | R100K, E107V & C580Y | cD11 | C580Y  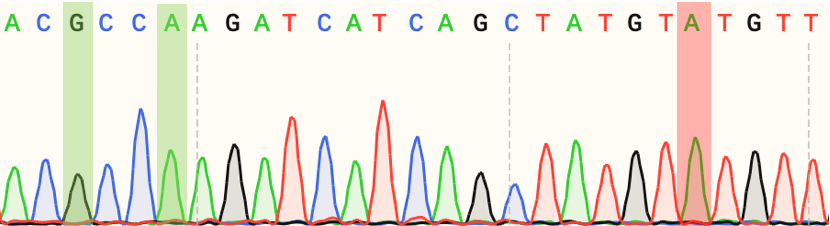    R100K, E107V  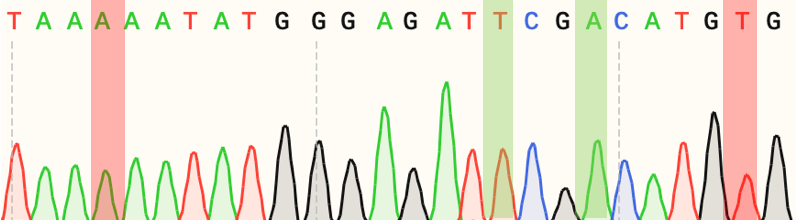 |
| Pikine_R | *Pf*Coronin | K100R, V107E | Revertant c1 | 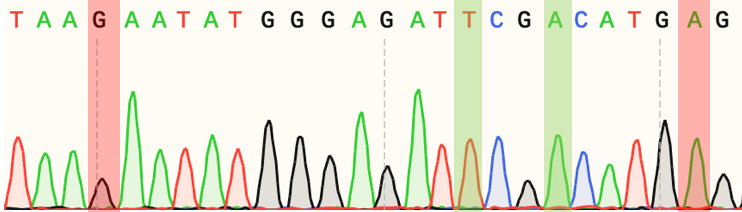 |
| Pikine_R | *Pf*Coronin | K100R, V107E | Revertant c2 | 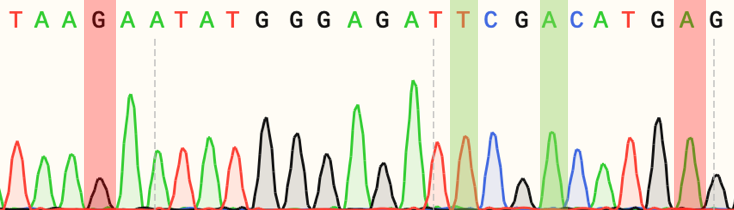 |
| Thiès |  | Wildtype |  | G50      I575  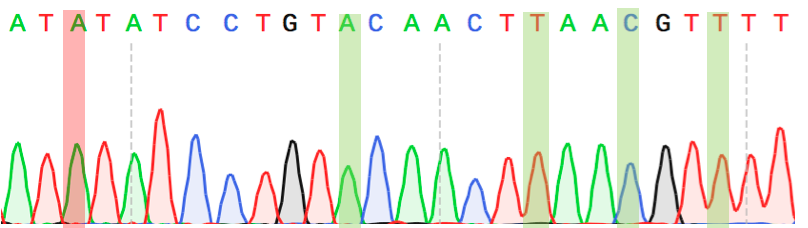 |
| Thiès_R | *Pf*Coronin | E50G | Revertant c1 | 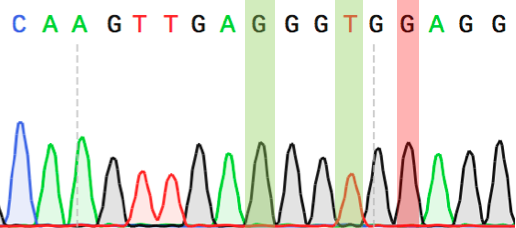 |
| Thiès_R | *Pf*Coronin | E50G | Revertant c2 | 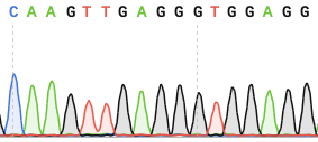 |
| Thiès_R | PF3D7_1433800 | M575I | Revertant c1 | 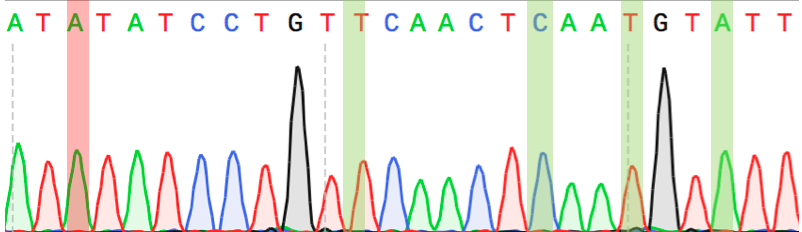 |
| Thiès_R | PF3D7_1433800 | M575I | Revertant c2 | 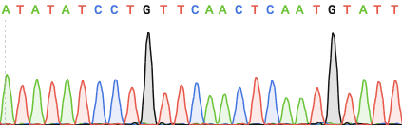 |
| 3D7 |  | Wildtype |  | R100, E107  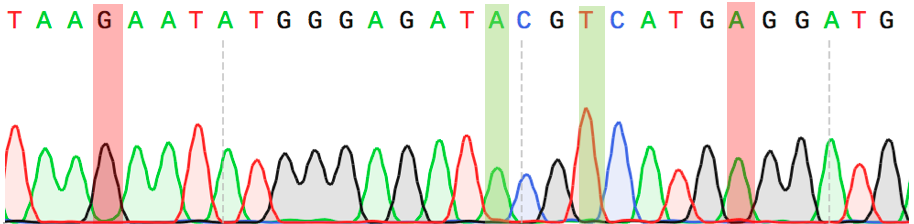    G50  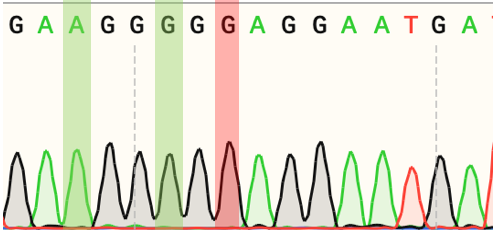    C580  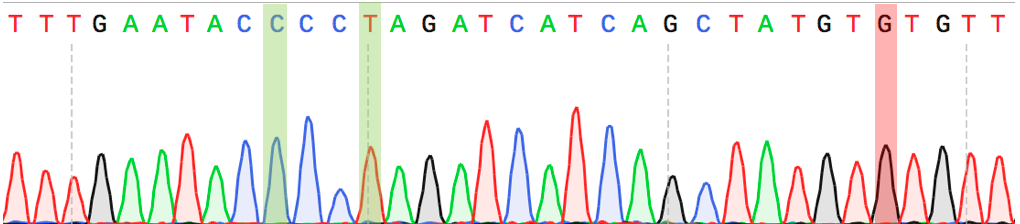    S1054  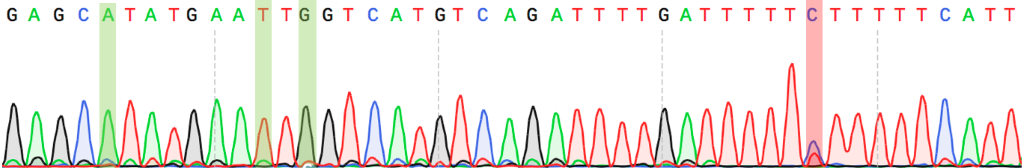 |
| 3D7 | *Pf*Coronin | R100K, E107V | cG6 | 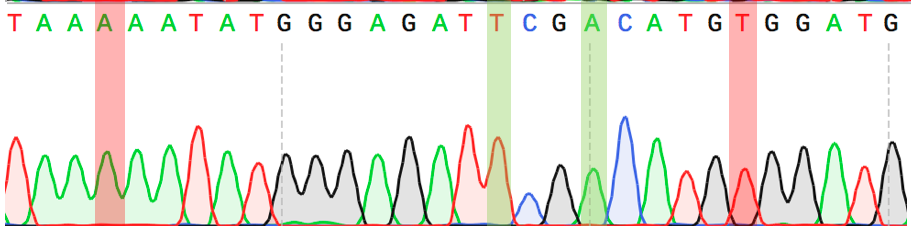 |
| 3D7 | *Pf*Coronin | G50E | cE11 | 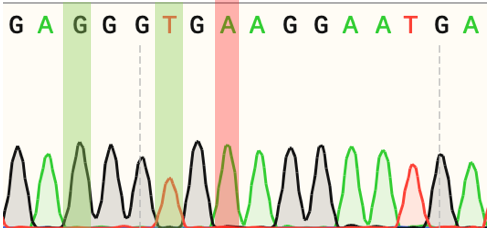 |
| 3D7 | *Pf*Kelch13 | C580Y | cB15 | 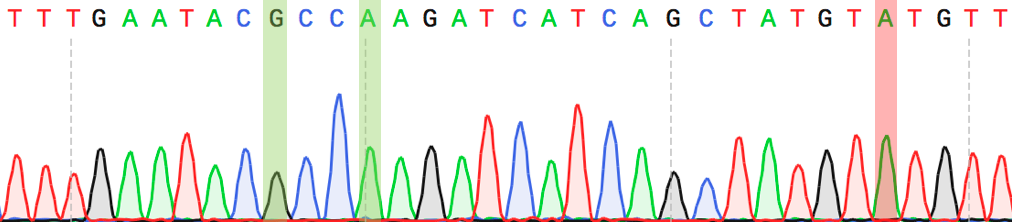 |
| 3D7 | PF3D7_1433800 | S1054F | c5 | 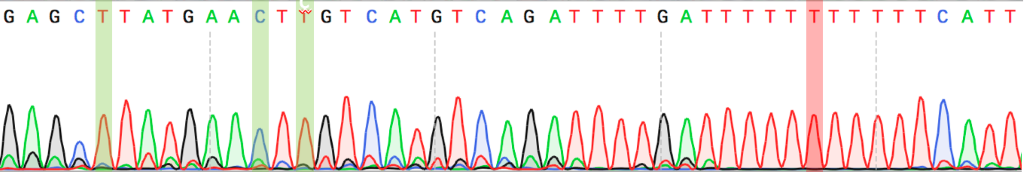 |
| 3D7 | PfCoronin & *Pf*Kelch13 | R100K, E107V & C580Y | cE9 | R100K, E107V  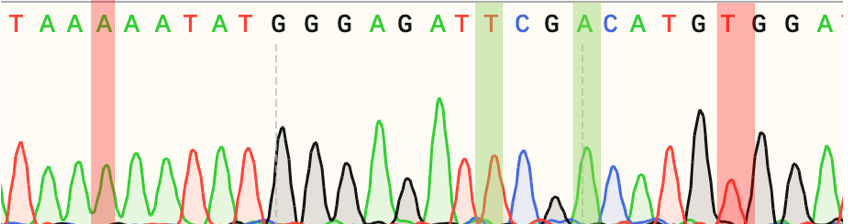    C580Y   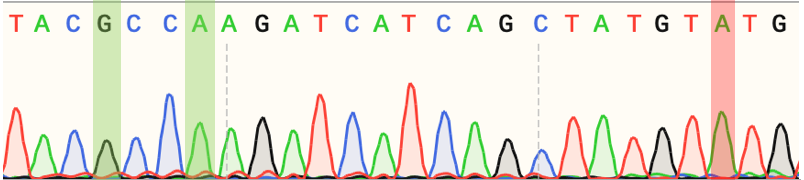 |
| 3D7 | *Pf*Coronin & *Pf*Kelch13 | R100K, E107V & C580Y | cG7 | R100K, E107V  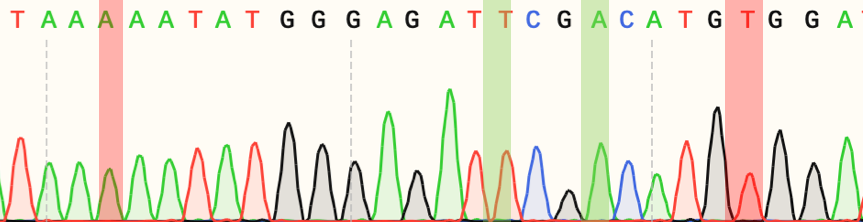    C580Y  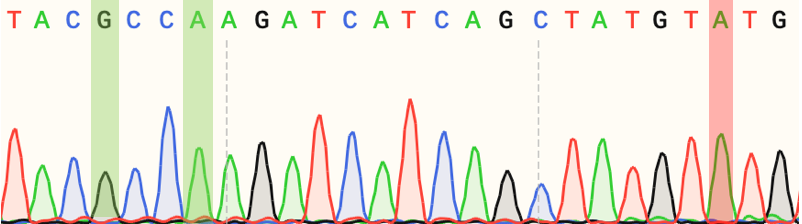 |
