# Supplementary material for: Genetic background and PfKelch13 affect artemisinin susceptibility of PfCoronin mutants in Plasmodium falciparum
Source: PLoS Genet. 2020 Dec 28;16(12):e1009266. doi: 10.1371/journal.pgen.1009266 (PMC7793257; doi:10.1371/journal.pgen.1009266)
Supplement: S3 Table — (DOCX) [file pgen.1009266.s013.docx]

| **Background** | **Protein** | **Mutation** | **Clone name** | **EC50, nM, Biorep1**  **(AM, DHA, MQ)** | **EC50, nM, Biorep2**  **(AM, DHA, MQ)** | **EC50, nM, Biorep3**  **(AM, DHA, MQ)** | **Mean EC50, nM**  **(AM, DHA, MQ)** | **StDev**  **(AM, DHA, MQ)** |
| --- | --- | --- | --- | --- | --- | --- | --- | --- |
| Pikine |  | Wildtype |  | 3.08, 2.67, 30 | 3.71, 1.08, 15.13 | 3.84, 3.48, 20.99 | 3.54, 2.41, 22.04 | 0.41, 0.99, 7.49 |
| Pikine | *Pf*Kelch13 | C580Y | cD5 | 3.76, 1.03, 29.07 | 1.92, 1.47, 31.13 | 1.99, 3.6, 31.30 | 2.56, 2.03, 30.50 | 1.04, 1.12, 1.24 |
| Pikine | *Pf*Kelch13 | C580Y | cE3 | 3.74, 1.10, 22.90 | 1.38, 1.34, 29.80 | 2.26, 3.92, 33.89 | 2.46, 2.12, 28.86 | 1.18, 1.28, 5.55 |
| Pikine | *Pf*Coronin & *Pf*Kelch13 | R100K, E107V & C580Y | cG9 | 3.59, 2.09, 26.12 | 1.98, 1.49, 30.15 | 2.23, 3.32, 30.97 | 2.60, 2.3, 29.08 | 0.87, 0.76, 2.60 |
| Pikine | *Pf*Coronin & *Pf*Kelch13 | R100K, E107V & C580Y | cD11 | 4.44, 3.05, 27.01 | 2.88, 0.94, 38.62 | 0.86, 2.5, 35.51 | 2.73, 2.16, 33.71 | 1.79, 0.89, 6.01 |
| Pikine_R | *Pf*Coronin | K100R, V107E | Revertant c1 | 5.57, 3.08, 21.89 | 2.59, 3.38, 34.26 | 3.96, 1.59, 18.70 | 4.04, 2.68, 24.95 | 1.49, 0.78, 8.22 |
| Pikine_R | *Pf*Coronin | K100R, V107E | Revertant c2 | 3.97, 2.89, 16.46 | 1.44, 1.65, 30.77 | 3.12, 1.44, 15.48 | 2.84, 1.99, 20.90 | 1.29, 0.64, 8.56 |
| Thiès |  | Wildtype |  | 7.26, 3.42, 26.22 | 8.52, 4.76, 37.44 | 6.74, 2.08, 17.38 | 7.51, 3.42, 27.01 | 0.92, 1.09, 10.05 |
| Thiès_R | *Pf*Coronin | E50G | Revertant c1 | 5.26, 2.59, 17.24 | 8.72, 5.00, 25.80 | 7.64, 1.51, 17.76 | 7.21, 3.03, 20.27 | 1.77, 1.46, 4.80 |
| Thiès_R | *Pf*Coronin | E50G | Revertant c2 | 9.09, 4.24, 24.69 | 7.94, 4.59, 35.03 | 5.31, 1.06, 15.62 | 7.45, 3.30, 25.11 | 1.94, 1.59, 9.71 |
| Thiès_R | PF3D7_1433800 | M575I | Revertant c1 | 12.94, 6.56, 41.11 | 11.94, 6.53, 52.35 | 5.44, 1.19, 21.12 | 10.11, 4.76, 38.19 | 4.07, 2.52, 15.82 |
| Thiès_R | PF3D7_1433800 | M575I | Revertant c2 | 11.54, 4.18, 42.40 | 10.39, 5.94, 55.01 | 7.65, 1.19, 27.69 | 9.86, 3.77, 41.70 | 1.99, 1.96, 13.67 |
| 3D7 |  | Wildtype |  | 2.54, 1.14, 18.99 | 1.18, 2.22, 14.31 | 2.17, 5.03, 19.75 | 1.96, 2.80, 17.68 | 0.70, 1.64, 2.95 |
| 3D7 | *Pf*Coronin | R100K, E107V | cG6 | 1.60, 5.42, 18.33 | 2.79, 2.71, 29.21 | 1.42, 8.54, 18.26 | 1.93, 5.56, 21.93 | 0.74, 2.38, 6.30 |
| 3D7 | *Pf*Coronin | G50E | cE11 | 2.78, 0.48, 11.28 | 1.35, 2.62,12.54 | 3.03, 5.3, 25.13 | 2.39, 2.8, 16.28 | 0.91, 1.97, 7.69 |
| 3D7 | *Pf*Kelch13 | C580Y | cB15 | 1.70, 1.12, 18.98 | 1.65, 1.11, 26.04 | 1.66, 1.16, 25.06 | 1.67, 1.13, 23.36 | 0.026, 0.022, 3.82 |
| 3D7 | PF3D7_1433800 | S1054F | c5 | 3.94, 2.53, 20.92 | 2.10, 1.54, 14.38 | 3.20, 2.33, 20.44 | 3.08, 2.13, 18.58 | 0.93, 0.43, 3.65 |
| 3D7 | PfCoronin & *Pf*Kelch13 | R100K, E107V & C580Y | cE9 | 18.75, 5.06, 30.54 | 10.67, 4.89, 24.60, | 33.47, 5.59, 45.65 | 20.96, 5.18, 33.60 | 11.56, 0.30, 10.85 |
| 3D7 | *Pf*Coronin & *Pf*Kelch13 | R100K, E107V & C580Y | cG7 | 21.25, 10.85, 30.08 | 16.95, 3.25, 21.51 | 34.67, 9.81, 46.42 | 24.29, 7.97, 32.67 | 9.24, 3.36, 12.66 |
