# Supplementary material for: Genetic background and PfKelch13 affect artemisinin susceptibility of PfCoronin mutants in Plasmodium falciparum
Source: PLoS Genet. 2020 Dec 28;16(12):e1009266. doi: 10.1371/journal.pgen.1009266 (PMC7793257; doi:10.1371/journal.pgen.1009266)
Supplement: S4 Table — (DOCX) [file pgen.1009266.s014.docx]

| **Position** | **REF (3D7)** | **ALT** | **Amino acid change** | **Parasite background** |
| --- | --- | --- | --- | --- |
| 1351050 | T | C | N2444D | Pikine and Thies |
| 1351257 | A | C | L2375V | Thies |
| 1353195 | T | C | N1729D | Pikine |
| 1354509 | C | T | V1291I | Thies |
| 1355219 | G | A | S1054F | ART Resistant line Pikine_R |
| 1355449 | G | A | synonymous | Pikine and Thies |
| 1355517 | C | T | G955R | Pikine |
| 1356284 | A | G | I699T | Pikine |
| 1356655 | T | C | I575M | ART Resistant line Thies_R |
| 1357486 | A | G | synonymous | Pikine and Thies |
| 1357558 | A | G | synonymous | Thies |
| 1357563 | G | A | H273Y | Thies |
| 1357565 | A | T | I272K | Thies |
