# Supplementary material for: Genetic background and PfKelch13 affect artemisinin susceptibility of PfCoronin mutants in Plasmodium falciparum
Source: PLoS Genet. 2020 Dec 28;16(12):e1009266. doi: 10.1371/journal.pgen.1009266 (PMC7793257; doi:10.1371/journal.pgen.1009266)
Supplement: S6 Table — For mutagenesis (mut) primers, shield mutations are highlighted in green and targeted mutation(s), if present within the range of the primer, are highlighted in red. (DOCX) [file pgen.1009266.s016.docx]

| Primer target | Primer name | Sequence 5'-3' |
| --- | --- | --- |
| PF3D7_1251200 | coronin G50E Fwd1 | GTATAGCTTGTAGTGCTGGATATATTG |
| PF3D7_1251200 | coronin G50E Rev1 | GGATTCCATGATAATATATTTACTTTTTTCTTATG |
| PF3D7_1251200 | coronin R100K & E107V Fwd2 | GGTGCGCTTTAAAGATTAATTC |
| PF3D7_1251200 | coronin R100K & E107V Rev2 | CTGACAAGTTCCACTTAATAAATTAC |
| PF3D7_1251200 | coronin sequencing Rev3 | CTCTCATCATAATGTGGTAATAAAGG |
| PF3D7_1251200 | coronin G50E mut revert fw1 | GGTACCATGGCAAGTTGAGGGTGGAGGAATGATCGGAGTTATC |
| PF3D7_1251200 | coronin G50E mut revert rv1 | CTGATAACTCCGATCATTCCTCCACCCTCAACTTGCCATGGTAC |
| PF3D7_1251200 | coronin G50E mut fw2 | GGTACCATGGCAAGTTGAGGGTGAAGGAATGATCGGAGTTATC |
| PF3D7_1251200 | coronin G50E mut rv2 | CTGATAACTCCGATCATTCCTTCACCCTCAACTTGCCATGGTAC |
| PF3D7_1251200 | coronin R100K & E107V mut revert fw1 | GTCTATAAGAATATGGGAGATTCGACATGAGGATGAGAATGTG |
| PF3D7_1251200 | coronin R100K & E107V mut revert rv1 | CTCATTCACATTCTCATCCTCATGTCGAATCTCCCATATTCTTATAG |
| PF3D7_1251200 | coronin R100K & E107V mut fw2 | GTCTATAAAAATATGGGAGATTCGACATGTGGATGAGAATGTG |
| PF3D7_1251200 | coronin R100K & E107V mut rv2 | CTCATTCACATTCTCATCCACATGTCGAATCTCCCATATTTTTATAG |
| PF3D7_1343700 | kelch13 C580Y Fwd1 | GTCAAATGGTAGAATTTATTGTATTGG |
| PF3D7_1343700 | kelch13 C580Y Rev1 | CTGCCATTCATTTGTATCTGGTG |
| PF3D7_1343700 | kelch13 C580Y Rev2 | GTTCAACGGAATCTAATATGTTATGTTC |
| PF3D7_1433800 | Unknown protein I575M Fwd1 | CCATACAAATGTCAAAATATAACAAACC |
| PF3D7_1433800 | Unknown protein I575M Rev1 | CTAGTTGCACGATTTTGATAATTTCC |
| PF3D7_1433800 | Unknown protein S1054F Fwd2 | GATAATGGTTTAAATTATAATATGTTGGAC |
| PF3D7_1433800 | Unknown protein S1054F Rev2 | CTAGTTGCACGATTTTGATAATTTCC |
| PF3D7_1433800 | Unknown protein I575M mut revert fw1 | ATATATCCTGTTCAACTCAATGTATTCCATTTTTATTATATGAAG |
| PF3D7_1433800 | Unknown protein I575M mut revert rv1 | GCTTCATATAATAAAAATGGAATACATTGAGTTGAACAGGATAT |
| PF3D7_1433800 | Unknown protein S1054F mut fw1 | GTAATTTATTAAATAGAGCTTATGAACTTGTCATGTCAGATTTTG |
| PF3D7_1433800 | Unknown protein S1054F mut rv1 | TCTGACATGACAAGTTCATAAGCTCTATTTAATAAATTACATTC |
